# Understanding the Formation of Heartwood in Larch Using Synchrotron Infrared Imaging Combined With Multivariate Analysis and Atomic Force Microscope Infrared Spectroscopy

**DOI:** 10.3389/fpls.2019.01701

**Published:** 2020-02-03

**Authors:** Sara Piqueras, Sophie Füchtner, Rodrigo Rocha de Oliveira, Adrián Gómez-Sánchez, Stanislav Jelavić, Tobias Keplinger, Anna de Juan, Lisbeth Garbrecht Thygesen

**Affiliations:** ^1^ Biomass Science and Technology Group, Department of Geosciences and Natural Resource Management, University of Copenhagen, Frederiksberg, Denmark; ^2^ Chemometrics Group, Department of Analytical Chemistry, University of Barcelona, Barcelona, Spain; ^3^ Nano-Science Center, Department of Chemistry, Faculty of Science, University of Copenhagen, Copenhagen, Denmark; ^4^ Section for GeoGenetics, Faculty of Health and Medical Sciences, Globe Institute, University of Copenhagen, Copenhagen, Denmark; ^5^ Wood Material Science Group, Department of Construction, Environment and Geomatics, Institute for Building Materials (IfB), ETH Zürich, Zürich, Switzerland; ^6^ WoodTec Group, Cellulose & Wood Materials, EMPA, Dübendorf, Switzerland

**Keywords:** heartwood formation, larch, extractives, synchrotron infrared imaging, Atomic Force Microscope Infrared Spectroscopy, Multivariate Curve Resolution – Alternating Least Squares

## Abstract

Formation of extractive-rich heartwood is a process in live trees that make them and the wood obtained from them more resistant to fungal degradation. Despite the importance of this natural mechanism, little is known about the deposition pathways and cellular level distribution of extractives. Here we follow heartwood formation in *Larix gmelinii* var. *Japonica* by use of synchrotron infrared images analyzed by the unmixing method Multivariate Curve Resolution – Alternating Least Squares (MCR-ALS). A subset of the specimens was also analyzed using atomic force microscopy infrared spectroscopy. The main spectral changes observed in the transition zone when going from sapwood to heartwood was a decrease in the intensity of a peak at approximately 1660 cm^-1^ and an increase in a peak at approximately 1640 cm^-1^. There are several possible interpretations of this observation. One possibility that is supported by the MCR-ALS unmixing is that heartwood formation in larch is a type II or *Juglans*-type of heartwood formation, where phenolic precursors to extractives accumulate in the sapwood rays. They are then oxidized and/or condensed in the transition zone and spread to the neighboring cells in the heartwood.

## Introduction

Heartwood (HW) formation is the final step in the life cycle of ray cells. Before cell death, ray cells undergo metabolic changes in the transition zone between sapwood (SW) and HW, resulting in increased synthesis of secondary metabolic compounds called extractives. The extractives have a significant effect on the properties of wood, most notably regarding its resistance to fungal decay and other forms of biological attack (Hillis, 1987; Hinterstoisser et al., 2000; Schultz and Nicholas, 2000; Taylor et al., 2002). In order to understand how extractives contribute to wood durability, many studies have focused on the chemical interplay between extractives and decay agents (Valette et al., 2017). However, there is an increasing understanding that the distribution of extractives within cell walls also plays an important role, though hitherto under-investigated (Kampe and Magel, 2013).

The process of HW formation has been studied for decades and is known to be associated with parenchyma cell death, disappearance of storage material, and increase in extractives content (Hillis, 1985; Hillis, 1987). Kampe and Magel (2013) described two possible HW formation mechanisms: Type I or *Robinia-Type* proposes the accumulation of the phenolic extractives in the transition zone without any indication of phenolic precursors in the aging SW (Nair et al., 1981; Magel et al., 1994; Bergström et al., 1999); Type II or *Juglans*-Type of HW formation suggests a gradual accumulation of phenolic precursors in the aging SW tissues. In type II, HW extractives are formed in the transition zone by primary and secondary reactions, such as oxidation and hydrolysis of precursor substances (Dellus et al., 1997; Burtin et al., 1998; Mayer et al., 2006). Once the extractives are formed, they are released into the lumina of neighboring cells and cell walls (Déjardin et al., 2010; Kampe and Magel, 2013). When inside the cell walls, a few studies suggest that at least some extractives are covalently bound to the structural cell wall polymers through enzymatic activity (Monties, 1991).

The extractive compounds typically associated with wood durability fall into one of several polyphenolic classes such as flavonoids, stilbenes, lignans, and polymers thereof. The types and quantities of these extractives are species dependent, genetically determined, and under environmental control (Hillis, 1987; Kwon et al., 2001; Taylor et al., 2002; Bito et al., 2011; Bush et al., 2011). In some species, extractives are present in lower amounts (e.g. Spruce 0.9-1.5%) (Willför and Holmbom, 2004) as compared to other species (e.g. larch up to 30%) (Gierlinger et al., 2004).

The genus Larix species (larch) are an important European resource for durable wood (Hillis, 1987). The extractives in larch belong to the molecular families of terpenoids, flavonoids, lignans, fatty acids, and galactans (Zule et al., 2015; Zule et al., 2017). Like all conifers, larch trees contain high amounts of oleoresin, produced by specialized epithelial cells surrounding resin canals and ray parenchyma cells. The resin is composed of fatty acids and esters thereof, as well as various subgroups of terpenoids, and is distributed throughout the HW and SW through the network of resin canals and ray cells (Hillis, 1987). It was shown for *Pinus sylvestris* that in HW, the composition of resin is enriched with phenolic compounds, presumably produced by ray parenchyma cells during HW formation (Felhofer et al., 2018).

Within the large family of terpenoids, diterpenoid acids (called resin acids) constitute the largest part of the oleoresin (Higuchi, 1997), and they have been repeatedly shown to have fungicidal properties (Keeling and Bohlmann, 2006), which is also the case for triterpenes, known as sterols (Burčová et al., 2018). Triglycerides and fatty acids may have a role in moisture regulation (Tomppo et al., 2011), which is important in the context of degradation by microorganisms. The principal phenolic compounds detectable in larch HW are flavonoids, the main compounds being taxifolin (C_15_H_12_O_7_) and dihydrokaempferol (C_15_H_12_O_6_). Minor amounts of lignans can also be found. Flavonoids have been attributed with fungicidal properties, but their main potential seems to be their ability to scavenge different types of radicals, as well as to reduce and chelate metals (Giwa and Swan, 1975; Cao et al., 1997; Babkin et al., 2001; Ivanova et al., 2012; Zule et al., 2017).

Larch HW is appreciated for its good mechanical properties, its color, and specially for its natural durability (Gierlinger et al., 2004). A strong relationship between extractives content and brown-rot decay resistance has been shown (Gierlinger et al., 2002; Windeisen et al., 2002). Nevertheless, very little is known about the formation and distribution of larch extractives within the xylem tissue at the cell and cell wall level. Their micro and nano-scale distribution is of importance (Taylor et al., 2002) since extractives are more effective against wood degradation within cell walls than in extracellular voids (Hillis, 1987). To investigate extractives in context with the microstructure, TOF-SIMS imaging has been applied in *Cryptomeria japonica* trees and showed that the extractives tend to accumulate near radial rays (Imai et al., 2005; Saito et al., 2008). Recently, the potential of Confocal Raman Microscopy to follow the extractive distribution in sapwood (SW) and heartwood (HW) of Scots pine (*Pinus sylvestris*, a moderately durable species) was shown (Belt et al., 2017; Felhofer et al., 2018). On the micro-level, pinosylvins were reported in the lumen, as well as in the compound middle lamella (CML), cell corner (CC), and pits of tracheid cells.

Synchrotron Radiation Fourier Transform Infrared (SR-FTIR) imaging is the ideal technique to study the extractive deposition patterns at the microscale during HW formation in larch because of the high brightness and high collimation of the beam and avoidance of the fluorescent problems experienced with Raman microspectroscopy. SR-FTIR imaging provides spatial and spectral information about the samples and, therefore, informs on the composition and location of the different sample constituents. Despite the relevant information contained in SR-FTIR images, the analysis of this kind of measurement is not straightforward because of the often large image sizes and the mixed signal components present in the spectra collected. To help in the signal-unmixing task, multivariate analysis tools like Multivariate Curve Resolution – Alternating Least Squares (MCR-ALS) are used. Indeed, MCR-ALS has already been proven to adapt particularly well to hyperspectral image analysis because of the easy introduction of external spectral and spatial information about the image in the analysis and the ability to work with both single and multiset (several) image structures (Tauler et al., 1995; de Juan et al., 2004; Piqueras et al., 2014). This approach is the main tool used in the current study to obtain distribution maps and spectral signatures of the wood sample (Felten et al., 2015; Piqueras et al., 2015).

To supplement the SR-FTIR images, atomic force microscopy infrared spectroscopy (AFM-IR) was used. AFM-IR combines atomic force microscopy (AFM) with pulsed IR laser (**Figure S1**) to obtain localized mid-IR spectra (3600-900 cm^-1^) of regions as small as tens of nm in the horizontal plane and with a vertical resolution of ~0.1 nm (Dazzi and Prater, 2017). Such resolution surpasses the resolution of optical IR instruments making AFM-IR a suitable technique to study nanoscale properties of wood materials. Within the study of plants, AFM-IR has been used to analyze the composition of thylakoids (Janik et al., 2013) and of epicuticular wax (Farber et al., 2019), and to understand how the structure and composition of the *Populus nigra* cell wall affects water transport within the xylem (Pereira et al., 2018). Further, it has been used to identify the products of the reaction between the cell wall of *Pinus taeda* and a phenol-formaldehyde resin (Wang et al., 2005). However, to our knowledge, no one has yet studied the nanoscale compositional variations between the cell wall, the middle lamella, and the rays of SW and HW.

The objective of this work was to obtain a detailed overview of the HW formation process in larch (*Larch gmelinii *i.e. var *Japonica)* at the micro and nanoscale by combining SR-FTIR, AFM-IR, and advanced chemometric tools.

## Materials and Methods

### Sample Preparation

**Figure 1:**
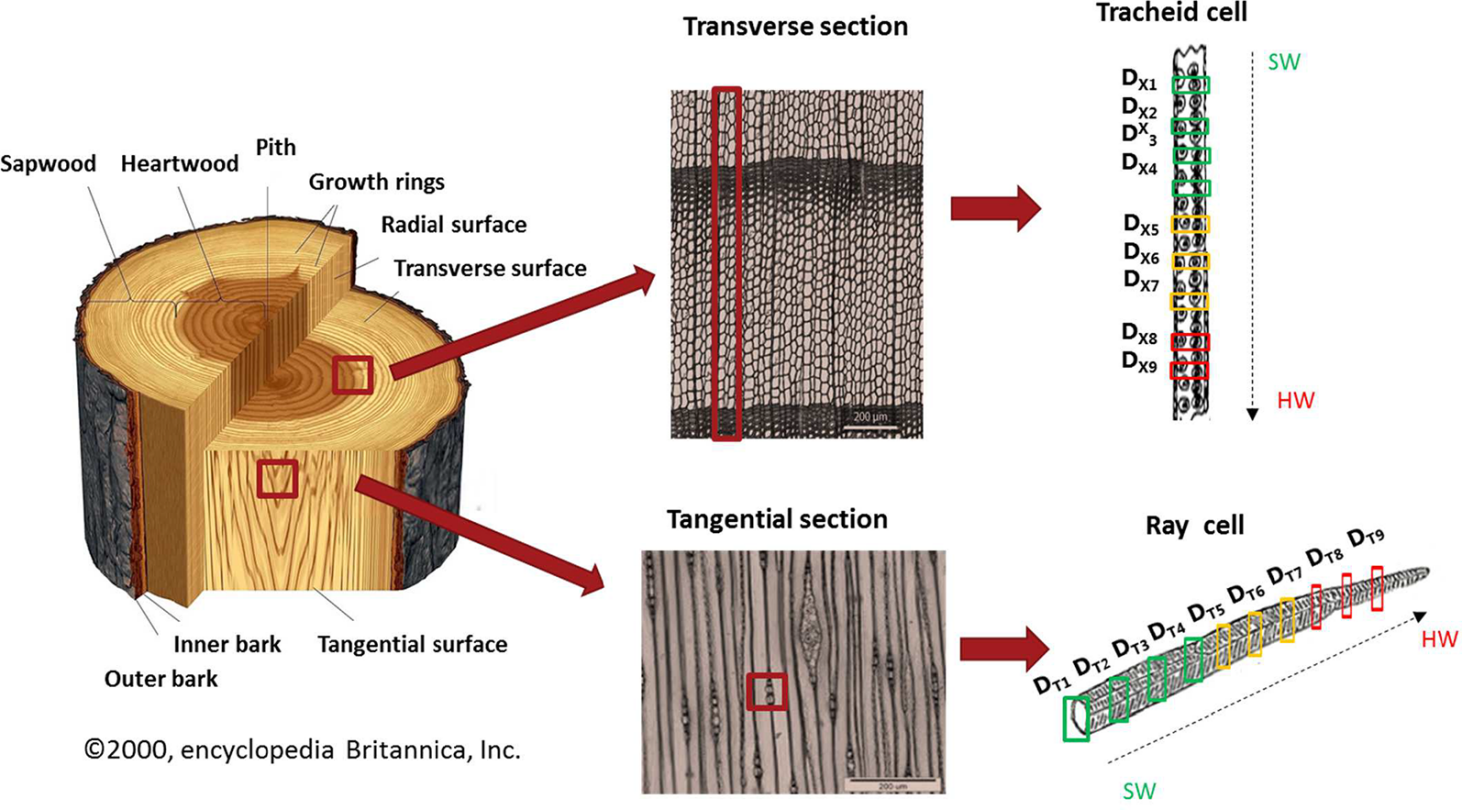
Representation of the sampling procedure. Left plot: Scheme of a tree trunk. Middle plots: Transverse and tangential micro-sections of wood (Photo: A. Musson/Royal Botanic Garden, Kew). The marked rectangles show the regions of interest for the heartwood formation study. Right plots: Collection of IR Synchrotron images (40 µm x 40 µm) of tracheid and ray cells of larch. The numbered rectangles mark the chosen annual rings (1-9) from sapwood to heartwood including the transition zone in between.

For this imaging study, a sample of *Larix gmelinii var japonica* (Kurile larch) was taken at 1.3 m stem height. The tree was felled in October 2017 near Hørsholm, north of Copenhagen, Denmark. The wood was freeze dried to avoid possible artifacts from air drying. From an area including nine annual rings, nine tangential (D_T1_-D_T9_) and nine cross-sections (D_X1_-D_X9_) were cut without any embedding of the samples, in order to preserve the major content and distribution of extractives. Samples of nominally 10 µm thickness were obtained using a Leica microtome (Leica RM2255) (**Figure 1**). For transportation, the samples were placed on a glass slide and covered with a glass coverslip.

### Synchrotron Infrared Imaging

All the tangential and cross-sections were imaged at the IR beamline MIRAS of the ALBA synchrotron (Cerdanyola del Vallés, Spain, proposal 2018022761). Before IR imaging, samples were inspected by light microscopy in order to select regions of interest (ROI) that included ray and tracheid cells and appeared to have a sample transparency that would allow SR-FTIR measurements in transmission mode. After area selection, the sample was carefully transferred onto a ZnSe disc of 1 mm thickness. The sample edges were fixed to the disc with tape to avoid sample movement during imaging. The IR measurements were acquired with a Bruker system (Hyperion 3000 microscope coupled to a Vertex 70 spectrometer) equipped with a liquid-nitrogen cooled mercury cadmium telluride (MCT) detector. Preliminary tests were carried out prior to the IR imaging. During these tests, possible effects of the high-energy IR beam on the wood material was studied by collecting punctual IR spectra at different exposure times. No adverse effects on the tissue or the spectra were observed for any of the exposure times tested. Therefore, we are confident that the energy of the IR beamline did not adversely influence our SR-FTIR measurements.

The IR images were acquired in transmission mode, using a 36x objective. The images were collected with a 3 x 3 µm^2^ spatial resolution. All spectra were obtained in the infrared region (4000−800 cm^−1^) with 64 co-added scans. Absorbance representation was used throughout. A total of nine SR-FTIR images were acquired in both tangential and cross directions.

### Atomic Force Microscopy Infrared Spectroscopy

AFM-IR exploits the photothermally induced resonance effect to detect the absorption of IR radiation with the AFM tip (Dazzi et al., 2005). In short, the sample is irradiated with the IR source and mechanically expands to dissipate the absorbed energy (**Figure S1**). The strongest expansion happens when the sample is irradiated with the IR wavelength that corresponds to the maximum absorption by the sample. Thus, by placing the AFM tip directly above the irradiated area, it is possible to detect the expansion of a sample by monitoring the deflection and oscillation of the AFM cantilever. This thermal expansion is directly proportional to the absorption coefficient of the excited area (Dazzi et al., 2012). By analyzing the many frequencies and amplitudes of the resonant oscillations of the AFM cantilever with Fourier transform, we extracted the useful information and reconstructed the IR spectra of various regions on the sample with a spatial resolution that is close to the size of an AFM tip.

We used a nanoIR from Analysis Instruments, Inc., to obtain mid-IR spectra of the ray, the middle lamella, and the secondary cell wall. Only two of the samples of the cross-sections (D_X1_ and D_X8_) were used for the AFM-IR investigation. We fixed the edges of a sample with adhesive tape to a glass slide and acquired AFM images in contact mode. First, we found a suitable region where we could see both the middle lamella and the tracheid cell wall, or the ray and the adjacent tracheid cell wall and imaged it. Then, we collected and averaged three-background IR spectra with the resolution of 4 cm^-1^ to account for the variations in power of the IR source. For the IR spectral acquisition, we chose the second mode of the cantilever vibration to record the signal. This mode had a frequency of about 190 kHz and we chose the frequency window to be ±25 kHz to account for the variations in thermoelastic properties between the cell walls and the middle lamella or the ray. The second mode was chosen to improve the signal to noise ratio of the cantilever amplitude. For AFM-IR, it is crucial to position the IR laser directly at the tip-sample contact to make sure that the recorded spectrum originates directly from the area at the sample above where the tip is positioned. To do so, we scanned around the tip-sample contact area with the IR laser to find the highest cantilever amplitude, which corresponds to the position where the IR laser has maximum power. Once the tip, sample, and laser positions were optimized, we collected the spectra at various positions of the sample with the energy resolution of 4 cm^-1^ and by co-averaging 256 scans. After IR spectral acquisition, the same area was inspected by using the build-in camera. No laser damage was observed on any of the samples. The AFM images were flattened to remove the tilt and the AFM-IR spectra were smoothed by a Savitzky-Golay filter (2^nd^ polynomial degree, 15 points window size) (Savitzky and Golay, 1964).

## SR-FTIR Data Treatment

The data treatment of SR-FTIR images consisted of two consecutive steps: (1) preprocessing of the image spectra to correct for scattering effects and (2) analysis by Multivariate Curve Resolution – Alternating Least Squares (MCR-ALS) (Tauler et al., 1995; de Juan and Tauler, 2006) to obtain pure spectra of the image constituents and their related distribution maps. The next subsections describe these steps.

### Data Preprocessing

Due to the thickness and density of the samples, the infrared spectra of ray and tracheid cells were oversaturated in both the low and the high spectral wavelength range. Therefore, these spectral areas had to be excluded, and only the 1200-1750 cm^-1^ range was included in the analysis.

Infrared spectra are prone to artifacts because of Mie scattering associated with surface irregularities. Such artifacts may produce a broad oscillation in the baseline spectrum and can lead to distortions in both the position and intensity of absorption bands (Romeo et al., 2006). The raw data were corrected by the algorithm Asymmetric Least Squares (AsLS) (Eilers, 2004), which has been demonstrated to cope well with this type of scattering (Piqueras et al., 2013).

### Hyperspectral Image Resolution

The goal of hyperspectral image resolution and, consequently, of the multivariate curve resolution alternating least squares (MCR-ALS) algorithm, is the decomposition of the original raw image data into distribution maps and pure spectra of the constituents present in the imaged sample (Tauler et al., 1995; Jaumot et al., 2005; de Juan and Tauler, 2006; Tauler et al., 2009). In the matrix form, hyperspectral images can be well described by a bilinear model based on the Beer-Lambert law [**Eq.(1)**], where the **D** matrix contains the original raw spectra, which are decomposed into a set of concentration profiles (**C** matrix) and corresponding pure spectra (**S** ^T^ matrix) of the constituents present in the image. Every row of the **S^T^** matrix corresponds to the pure spectrum of an image constituent, while every column of the **C** matrix of concentration profiles corresponds to the related pixel-to-pixel variation of its chemical concentration. It should be pointed out that each column of the **C** matrix can be refolded appropriately in order to recover the original two-dimensional spatial image structure and then pure distribution maps are obtained.

(1) $$D=CS^{T}+E$$

**Figure 2:**
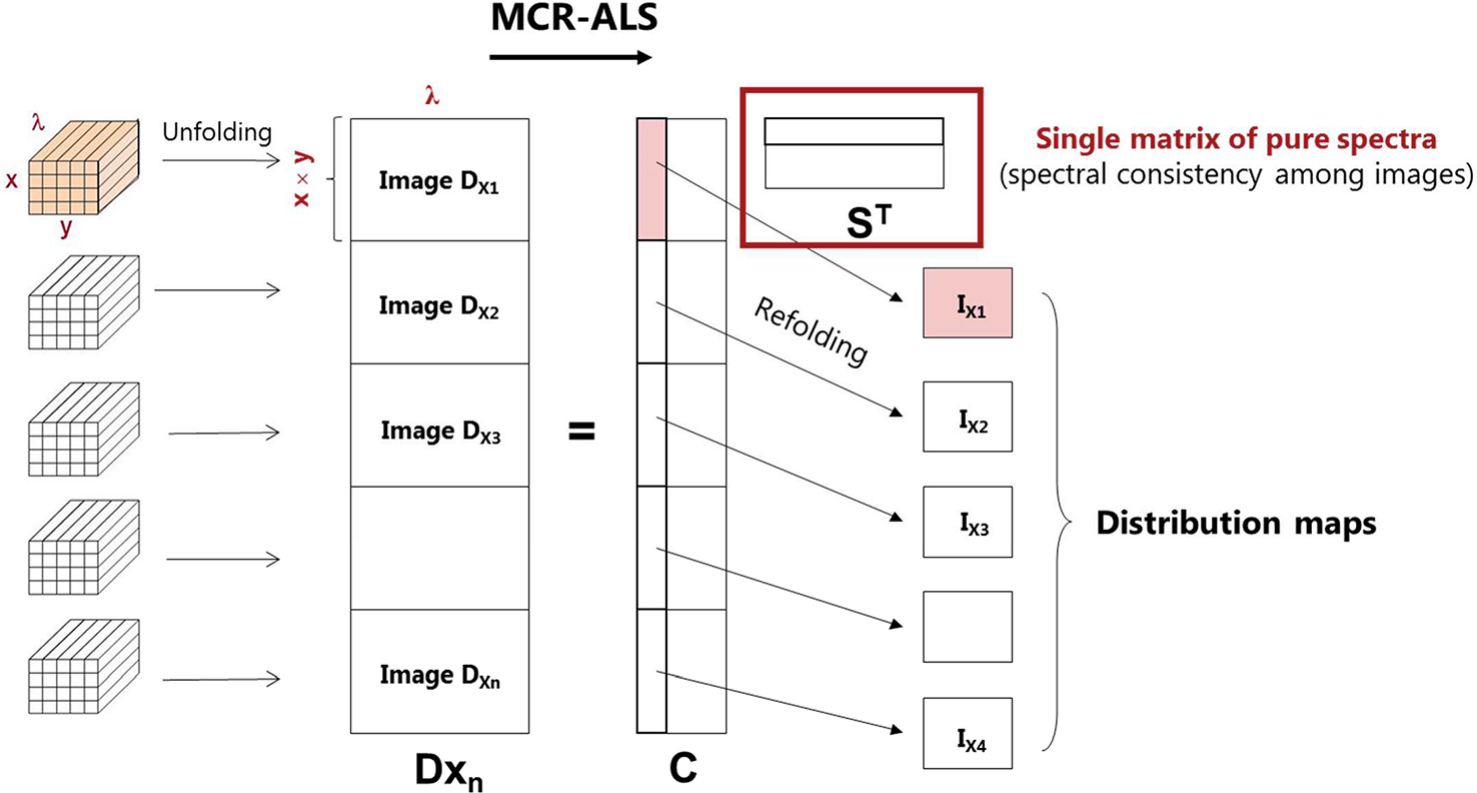
MCR-ALS analysis for a multiset structure of images, where x and y are spatial pixels and λ represents the wavenumbers of the spectra. **Dx_n_** is the augmented data matrix; **C** is the concentration profiles; and **S^T^** is the pure spectra matrix.

MCR-ALS is a flexible method that allows analyzing a single image individually or several images simultaneously. To obtain a complete and reliable description of the HW formation, the simultaneous analysis of the recorded images along the nine-annual rings obtained from larch sample was performed. Thus, two multisets were built and analyzed separately—formed by nine and eight images collected in the cross-sectional (**D**_X_) and tangential directions (**D**_T_), respectively. See **Figure 2** for a visualization of the image multiset structure. In these multisets, the spectral dimension of all images is the same, while the image dimensions may differ between images because the images are unfolded before being merged into a single matrix (**Figure 2**). Due to extreme cases of over-saturation, the first image of the cross-sections (**D**_T1_) and the last image of the tangential sections (**D**_X9_) had to be excluded from the multiset structures **D**_T_ and **D**_X_, i.e:

$$D_{\text{X}}=[D_{\text{X}1};D_{\text{X}2};D_{\text{X}3};D_{\text{X}4};D_{\text{X}5};D_{\text{X}61};{\text{D}}_{\text{X}62}D_{\text{X}7};D_{\text{X}8}]$$

$$D_{\text{T}}=\left[D_{\text{T}2};D_{\text{T}3};D_{\text{T}4};D_{\text{T}5};D_{\text{T}6};D_{\text{T}7};D_{\text{T}8};D_{\text{T}9}\right]$$

A multiset structure also follows the bilinear model based on Beer–Lambert law [see **Eq. (1)**]. In this example, image multiset analysis by MCR-ALS provides a single matrix **S^T^** of pure spectra, identical for all the images analyzed, and a **C** matrix formed by as many submatrices as the number of images included in the data set. Every column of each **C** submatrix can be refolded conveniently to recover the distribution map of each constituent present in the different images of the data set (**Figure 2**).

MCR-ALS multiset analysis was performed on both multiset structures **D**_X_ and **D**_T_ following the MCR-ALS steps described in the literature (Jaumot et al., 2005). The first step consisted of determining the number of components involved during HW formation by singular value decomposition of the whole preprocessed **D**_X_ and **D**_T_ matrices (Golub and Reinsch, 1970). Five contributions were needed to describe the variation in both multisets. Then, initial estimates of pure spectra were obtained with a method based on SIMPLISMA (Windig and Guilment, 1991). The spectral estimates and the original multiset are used to perform an iterative alternating least squares optimization of matrices **C** and **S** ^T^ under constraints. To obtain unmixed resolved profiles that are chemically meaningful, the constraints used in the resolution of both multiset structures were non-negativity in both, the concentration and the spectral profiles (Bro and de Jong, 1997), and normalization of pure spectra in the **S** ^T^ matrix (using 2-norm, i.e., the Euclidean norm).

After a preliminary MCR-ALS analysis of both multisets (**D**_X_ and **D**_T_) under non-negativity constraints, we realized by inspection of the distribution maps obtained that there were components absent in some sub-images of both multisets. As a consequence, a new MCR-ALS analysis was performed to obtain more accurate solutions by imposing the additional constraint of correspondence of species, which encodes information on the presence/absence of constituents in the concerned images of the multisets (Tauler et al., 2009), i.e. when a certain constituent is absent in one image, the related concentration profile is null. The lack of fit was 6.37% and 7.44% for **D**_X_ and **D**_T_, respectively, which is satisfactory for FTIR measurements (Felten et al., 2015).

It is important to note that, when resolving images of biological samples, each resolved contribution (component) may refer to a mixture of chemical compounds of defined composition (polysaccharides, sugars, polymers, fatty acids, flavonoids…) that are present in a particular location of the sample and often represent a distinct biological element, e.g., a tissue or a cell part. This means that an MCR contribution is not necessarily a pure chemical compound. In imaging, the component discriminating ability of MCR will also depend on how fine the spatial resolution of the imaging technique used is, e.g., if two components are differently distributed at nanoscale level, MCR will not resolve them if imaging is performed at microscale level.

## Results

### Exploratory Analysis of the SR-FTIR Images

**Figure 3:**
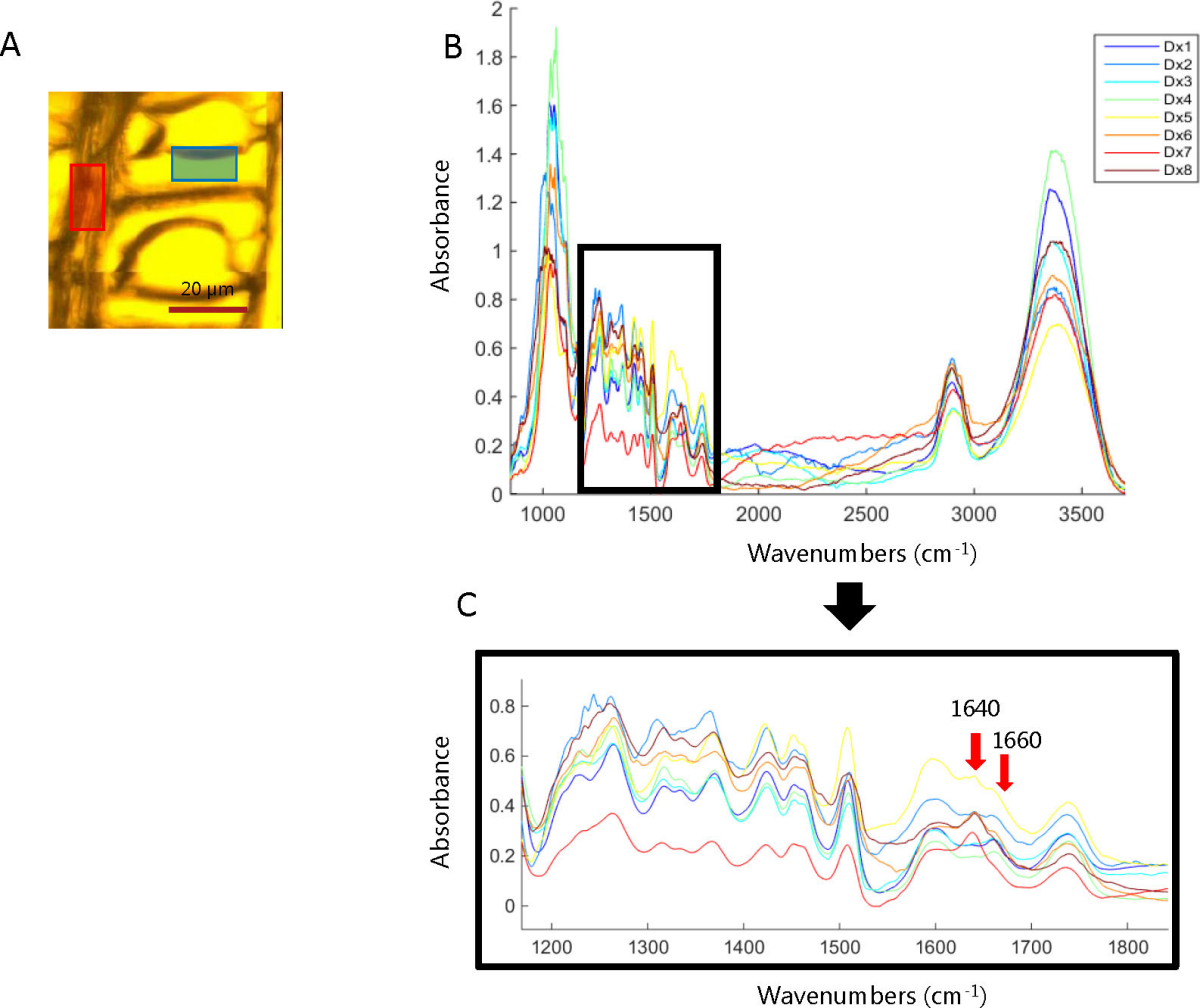
**(A)** Representation of the area selection of the lumen, cell wall, and ray. **(B)** Average spectra of the ray area selected for each of the cross-section images collected across the heartwood formation zone. **(C)** Zoom of the spectral range from 1200 cm^-1^ to 1800 cm^-1^ of the average spectra of the ray area selected for each of the cross-section images. The spectra gradually change from blue (sapwood) to red (heartwood) color.

In order to identify the main spectral variations during the HW formation of Kurile larch, an exploratory analysis was done. A small area of the ray and tracheid cell wall was selected for each of the images collected in the cross-sectional direction (**Figure 3A**). **Figure 3B** shows the average spectra of the ray area selected for each of the collected images (the average spectra of the tracheid cell wall area can be found in the supplementary information (**Figure S2**). The main spectral features when going from the SW to the HW of the ring area are the emergence of a new band at 1640 cm^-1^ and the decrease in intensity of the band at 1660 cm^-1^. These spectral features appear in the sample **D**_X5_, which we therefore determined to be the transition zone between SW and HW (**Figure 3C**). According to the literature, the band at 1640 cm^-1^ could correspond to adsorbed water (Popescu et al., 2007) or to a carbonyl stretching (C = O) as a consequence of the presence of para substituted ketone or aryl aldehydes (Lawther et al., 1996; Shi et al., 2012). Since the OH band at 3360 cm^-1^ appears not to co-vary with the band at 1640 cm^-1^, we find it unlikely that the latter is related to water adsorption.

### Resolution of Image Multiset Structures. MCR-ALS on Complete SR-FTIR Images

**Figure 4:**
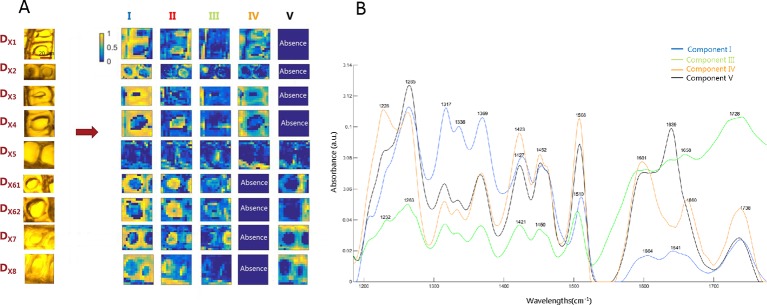
MCR-ALS image multiset results of the multiset structure formed by complete cross-section images. **(A)** Distribution maps of components involved in the heartwood formation of Kurile larch. Each line of maps represents the resolved maps of all constituents for a particular sample. Each column of maps represents the distribution map of a particular constituent in all samples analyzed. Distribution maps use a gradual color scale where yellow color refers to high concentration values and the blue color to low values. **(B)** Related pure spectra.

MCR-ALS multiset analysis on complete images provides the biological spectral signatures and distribution maps needed to integrally describe the ray and tracheid cells across the transition zone during the HW formation of larch. Although, as mentioned before, there is not necessarily a one-to-one correspondence between the MCR-ALS contribution and the individual chemical compounds, each contribution can be associated with some particular kind(s) of plant cell region, because of the morphology of the distribution maps and the spectroscopic features found in the resolved spectra. The resolution results for the multiset of the cross sections (**D**_X_) are shown in **Figure 4**. The multiset analysis of the tangential section images (**D**_T_) gave similar MCR-ALS results as **D**_X_ and are shown in **Figure S3**. The light microscopy images, corresponding to the imaged ray and the two surrounding tracheid cells areas are shown in the left of **Figure 4A**. The resolved distribution maps of each image are displayed in the right side of **Figure 4A** and the related resolved spectra in **Figure 4B**.

MCR-ALS was able to resolve the main plant cell constituents of the wooden tissue and to give further details on chemical changes occurring in the transition from SW to HW. Component I is characterized by signals in the region between 1317-1370 cm^-1^, which are mainly associated with cellulose (Colom et al., 2003; Popescu et al., 2010) (see **Table 1**). The corresponding distribution maps show higher intensity in what we identify as the secondary cell wall (S2) of the tracheids, known to be thick in latewood and rich in cellulose (Fengel and Wegener, 1989). This component was distributed evenly across all the growth rings, as is also seen clearly in **Figure S3A** of the tangential sections.

**Table 1:** The characteristic bands in FT-IR spectra of the studied samples and their assignments according to the literature data.

Wavenumber (cm^-1^); literature	Band origin (assignment) with comments	Wavenumber (cm^-1^) from SR-FTIR & AFM-IR; this work
**1060–1015**	C-O valence vibration mainly from C3-O3H (Cellulose)^a^; 1060cm^-1^ polysaccharide^b^	1056
**1072**	C-O deformation in secondary alcohol of galactosyl subunits^c^	1072–1076
**1095**	C-C and C-O stretching motions (cellulose)^a^	1096
**1108**	COH in plane deformation (celluloses and hemicelluloses)^d^; Aromatic C-H in plane deformation (typical syringyl units)^e^; C = O stretch^e^	1108
**1162-1125**	C-O-C valence vibration (polysaccharide)^b^; 1165cm^-1^ (characteristic for 5,7-dihydroxysubstituted flavonoids)^f^	1156–1164
**1230-1221**	C-C plus C-O plus C = O stretch^a^; lignin, Guaiacyl condensed > Guaiacyl etherified^g^	1220–1228
**1235-1225**	OH plane deformation, also COOH^a^	1232
**1270-1266**	Guaiacyl ring plus C = O stretch^a^	1263–1265
**1280-1277**	CH-deformation in cellulose^d^	1272–1284
**1317-1315**	CH_2_ rocking vibration (cellulose)^b^	1315–1317
**1335-1320**	CH in plane bending in cellulose^d^	1320–1332
**1365-1335**	OH plane deformation vibration (cellulose)^a^	1336
**1375-1365**	CH bending in cellulose^d^	1368–1372
**1430-1422**	CH_2_ scissoring in lignin and cellulose^d^ CH bending; aromatic skeletal vibrations with C-H plane deformation in lignin^a^	1423–1432
**1460**	Asymmetric C-H bending from methoxy groups in lignins^a;h^; asymmetric C-H bending in CH_3_ and CH_2_ in pyran for hemicelluloses^h^	1452–1456
**1520-1505**	Aromatic skeletal vibrations in lignin^a^	1520–1505
**1605-1593**	Aromatic skeletal vibrations plus C = O stretching in lignin^a^	1595–1608
**1640-1635**	C = O stretching of para substituted ketone or aryl aldehydes^i^; C = O stretch of taxifolin^j^	1640–1635
**1650-1640**	H-bonded C = O stretching in coniferyl/sinapyl aldehyde ^k^	1640–1648
**1655-1660**	Ring conj. C = C stretching of Coniferyl/sinapyl alcohol; C = O stretch of coniferyl/sinapyl aldehyde^k^	1660
**1690-1670**	C = O stretching in conjugated ketones^h^	1672–1692
**1700-1690**	C = O vibration in carboxylic group in resin acid ^h^	1700–1690
**1730-1725**	C = O vibration of acetyl-or COOH- groups ^a^	1728
**1738-1709**	C = O stretching in unconjugated ketones, carbonyls and ester groups (frequently of carbohydrate origin)^a,e^	1724–1740
**1770-1760**	C = O stretching in conjugated ketones^e^	1752–1764

^a^ Schwanninger et al., 2004; ^b^ Fackler et al., 2010; ^c^ Ghosh et al., 2015; ^d^ Popescu et al., 2010; ^e^ Popescu et al., 2007; ^f^ Zu et al., 2012; ^g^ Faix, 1991; ^h^ Traoré et al., 2018; ^i^ Lawther et al., 1996; ^j^ Kocábová et al., 2016; ^k^ Bock and Gierlinger, 2019.

The most prominent bands of lignin at 1505 and 1610 cm^-1^ are associated with C = C stretching of the aromatic ring modes (Colom et al., 2003; Weiland and Guyonnet, 2003; Popescu et al., 2010; Gorsás et al., 2011). They appear in components I, III, IV, and V, but show higher intensity and thus higher lignin contribution in components IV and V. Components IV and V appear to be situated in the same anatomical segments, i.e. in the cell corners (CC) and in the compound middle lamella (CML; middle lamella + adjacent primary walls), which is consistent with previous studies showing high lignin concentration in these locations (Fengel and Wegener, 1989). The main spectral difference between component IV and V is the same spectral variation that was observed during the exploratory analysis of SR-FTIR images: Component IV shows the characteristic band at 1660 cm^-1^, which is attributed to the ethylenic C = C (in coniferyl alcohol/sinapyl alcohol units) and C = O (in coniferaldehyde/sinapaldehyde) bond stretches of lignin (Umesh and Rajai, 2010; Umesh et al., 2011; Bock and Gierlinger, 2019). This component was prevalent in the CC and CML of SW tracheid cells but disappears in the HW. The other feature observed in the preliminary analysis, the band at 1640 cm^-1^, only appears in component V, which is distributed in the CC, CML, and part of the ray area of HW tracheids, as is also observed in **Figure S3A**. According to the literature, the IR band at 1640 cm^-1^ is assigned to a carbonyl stretching due to para substituted ketone or aryl aldehydes (Lawther et al., 1996; Shi et al., 2012). This band was also assigned to hydrogen bonding to the carbonyl group, as reported elsewhere (Pomar et al., 2002; Agarwal and Reiner, 2009; Bock and Gierlinger, 2019). Because component IV is present from **D**_X1_ to **D**_X5_ (SW region), and component V appears from **D**_X5_ to **D**_X8_ (HW region), it can be deducted that this fifth annual ring represents the transition zone, where the process of HW formation starts.

The resolved IR spectrum for component III shows a distinct, broad band at 1700-1736 cm^-1^ and is assumed to be formed by the overlapping of the C = O stretch vibration of acetyl or carboxylic acid (COOH) groups (Faix, 1991; Schwanninger et al., 2004) and the C = O stretch vibration of unconjugated ketones, carbonyls, and esters groups. This broader band centered at 1728 cm^-1^ could suggest the presence of resin acids in the rays since C = O stretching belonging to the -COOH group absorb around 1700 cm^-1^ (Traoré et al., 2018). Besides, bands are observed around 1600, 1637, and 1660 cm^-1^; the same ones as found in components IV and V, but weaker. Candidates for para substituted ketones are flavonoids such as taxifolin and dihydrokaempferol (Ruddick and Xie, 1994; Kocábová et al., 2016), known to be abundant in larch (Nisula, 2018). This component is represented in all the images in the ray, as well as tracheid lumina and S3 layer.

As mentioned before, resolved IR spectra reflect a mixture of different kinds of biomolecules. For example, the IR spectrum related to component IV consists of a mixture of mainly lignin and hemicelluloses, known by the presence of the band at 1738 cm^-1^, which is attributed to ester carbonyl groups prominent in hemicelluloses (Stewart et al., 1995; Gorsás et al., 2011) (see **Table 1** for the different IR bands of wood assembled from the literature). Finally, since the samples were measured in the dry state for the SR-FTIR experiment, the IR spectrum of component II associated with part of the lumen is not shown, since it does not contain any biologically relevant information. The spectrum corresponds to the IR absorbance of the ZnSe slide used in the measurements.

### AFM-IR Spectra Analysis

**Figure 5:**
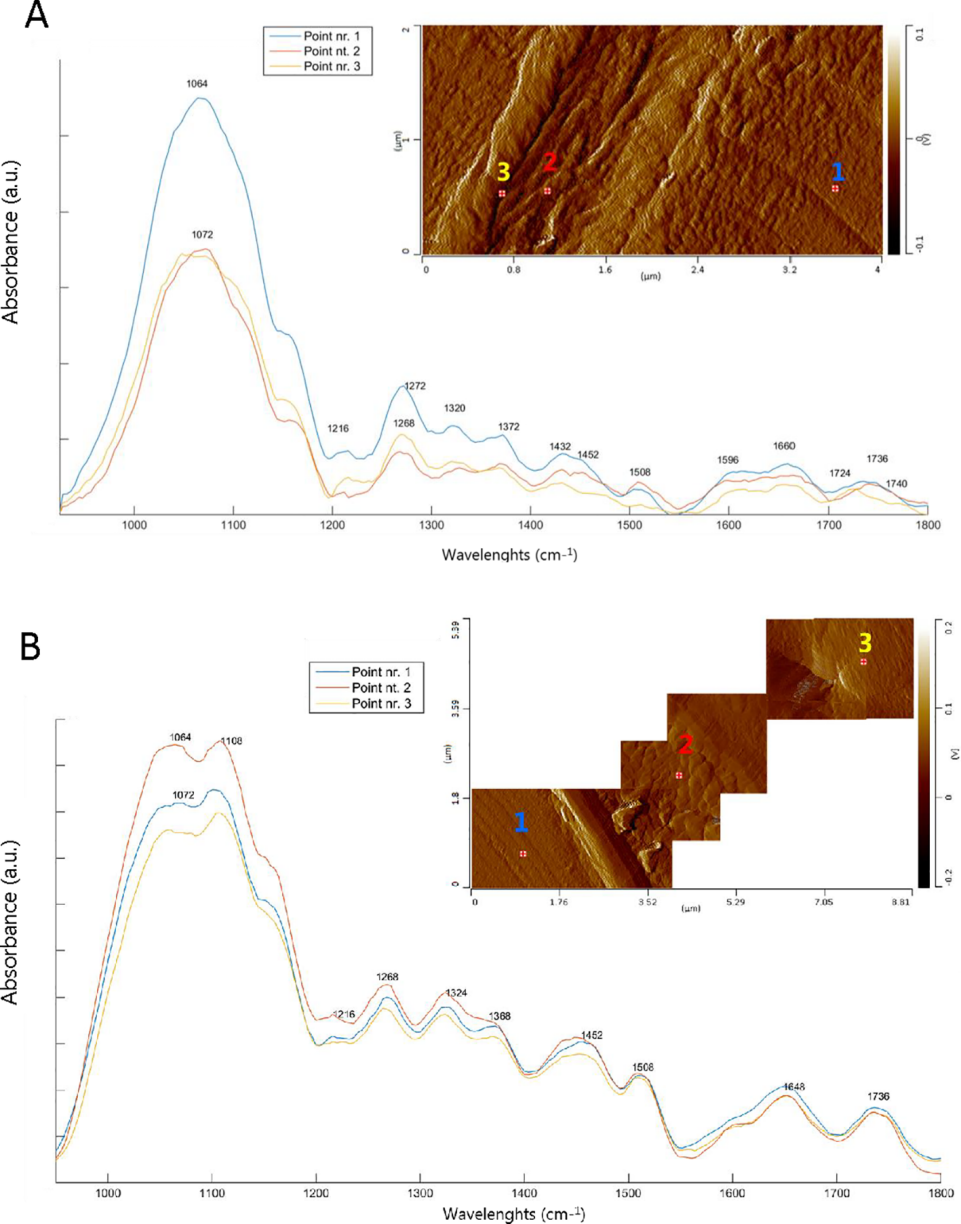
AFM deflection images and AFM-IR spectra of the middle lamella and secondary cell wall. The numbered crosses on the AFM images indicate the location of the AFM-IR spectra. Point nr.1: Secondary cell wall (S2); point nr.2: Compound middle lamella (CML) and point nr.3: Assumable primary cell wall. **(A)** sapwood tracheid cell (**D**_x1_) and **(B)** heartwood tracheid cell (D_x8_).

The AFM-IR spectra collected in the tracheid cell in the cross-sectional sections of SW (**D**_X1_) and HW (**D**_X8_) are shown in **Figure 5**, together with the AFM images. Where possible, we acquired the AFM image of the middle lamella and cell wall in a single image (**Figure 5A**). However, in some places, the middle lamella (and the ray) contain topographical features that exceed a few micrometers. Such complex topography is difficult to image in contact mode because of the limited vertical range of the AFM scanner. In addition, the imaging of such complex topographical features blunts the AFM tip rapidly. Hence, we took images of various dimensions, depending on the topography of the area, in order to minimize the mechanical strain exerted on the AFM tip, but to still be able to get a view of both the cell wall and the middle lamella in the same image (**Figure 5B**). Numbered crosses on the AFM images indicate the location of AFM-IR spectra. In contrast to the SR-FTIR spectra, the spectral region between 1000-1200 cm^-1^ is not saturated in the AFM-IR measurements, so we were able to obtain information from that region as well. Although the IR spectra of CML (point nr. 2) and S2 layer (point nr. 1) are very similar to each other in the SW (**Figure 5A**), we can observe different intensities around 1504-1510 cm^-1^, associated with C = C stretching of the aromatic ring modes of lignin. This band is more intense in the CML, which was also seen in the SR-FTIR data and is consistent with literature (Fengel and Wegener, 1989). The C = O stretching mode is slightly shifted to lower wavelengths (1724 cm^-1^) in point nr. 3, which is characteristic of pectins and/or hemicelluloses (Gorsás et al., 2011). Cellulose bands dominate the IR spectra in the S2 layer (see **Table 1**). In the case of the HW tracheids (**Figure 5B**), no spectral differences between the CML (point nr. 2) and S2 layer (point nr. 1) were observed. By comparing AFM-IR spectra of **Figures 5A** (SW) and **B** (HW), we see the presence of the band at 1648 cm^-1^, while the band at 1660 cm^-1^ is missing in the HW tracheid cell wall; the opposite is the case for SW. These are the same main spectroscopic features that were found in the analysis of SR-FTIR images. Finally, a new band at 1108 cm^-1^ appears in HW (**Figure 5B**), linked with COH in plane deformation of celluloses and hemicelluloses (Schwanninger et al., 2004) and/or with aromatic C-H in plane deformation of lignin.

**Figure 6:**
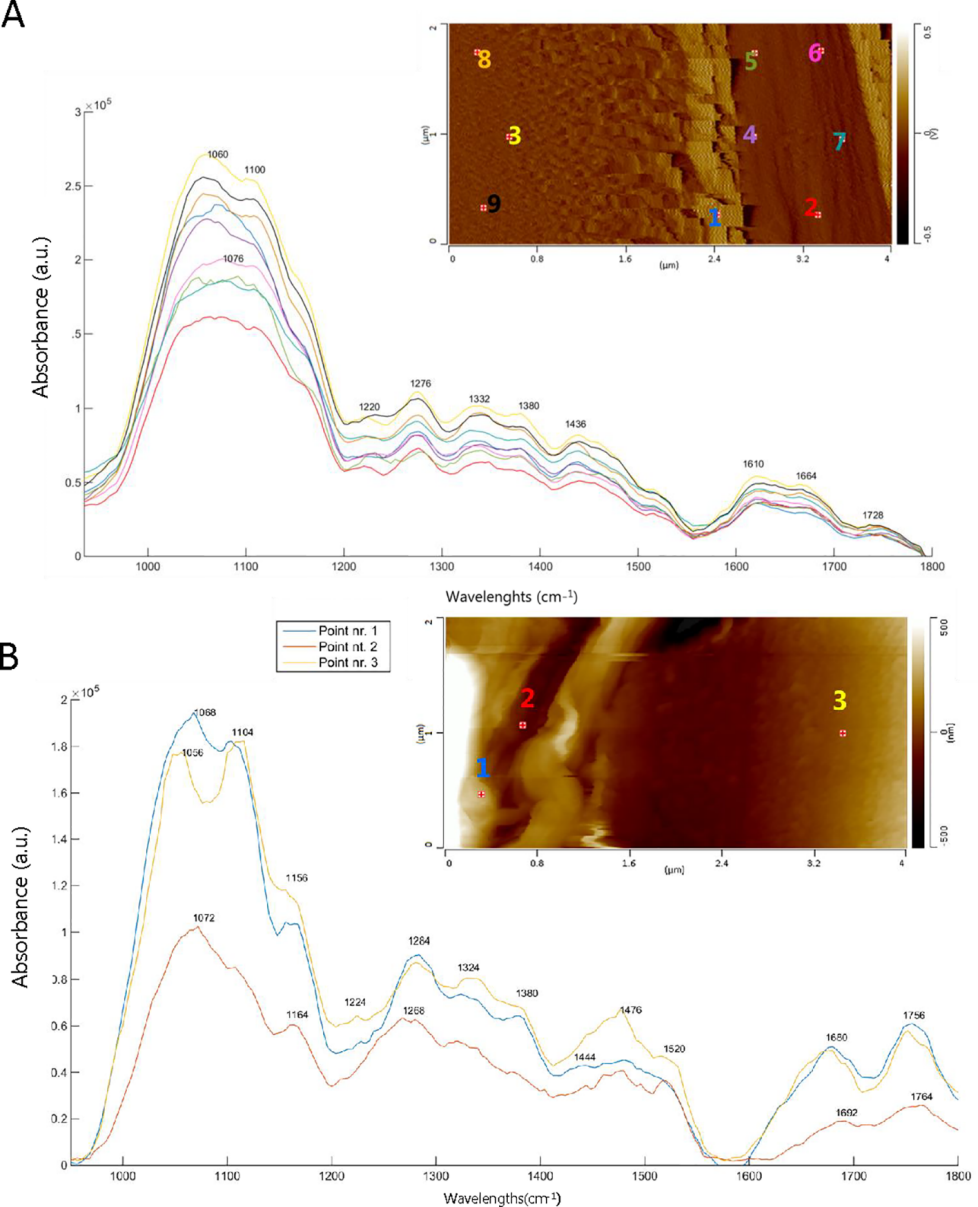
AFM deflection (a) and height (b) images and AFM-IR spectra of the ray cell and secondary cell wall. The numbers on the AFM images indicate the location of the AFM-IR spectra. **(A)** sapwood tracheid and ray region (**D**_x1_) where points nr.: 1, 2, 4, 5, 6, 7 correspond to the ray and points nr.: 3, 8, 9 to the secondary layer (S2) of tracheid cell wall. **(B)** heartwood tracheid and ray region (D_x8_) where points nr.: 1, 2 are the analyses of the ray and point nr. 3 of the secondary layer (S2) of tracheid cell wall.

**Figure 6** shows the AFM images and AFM-IR spectra of the ray region and the S2 cell wall of an adjacent tracheid in SW (**Figure 6A**) and HW (**Figure 6B**). Imaging of the ray and the cell wall in contact mode was particularly difficult because of their different material properties. This is why the image is blurry and it appears as if the ray is smeared over the cell wall (**Figure 6**). Such topography and relationship between the ray and the cell wall are unrealistic and simply an artefact of imaging in contact mode. This artefact does not affect the acquisition of AFM-IR spectra or its spectral features because the spectra are acquired after imaging was finished and at the frequency characteristic for the tip-substrate system. Lignin and cellulose bands are more intense in the S2 cell wall (points nr. 3, 8, and 9) of SW compared to the ray area (points nr. 1, 2, 4, 5, 6, and 7). A characteristic peak occurs at 1076 cm^-1^ inside the ray, assigned to C-O bands in primary and secondary alcoholic groups (Compounds and Hergert, 1960). A low intensity of the band at 1728 cm^-1^ is seen in the S2 cell wall. The ray region spectrum of HW (points nr.1 and 2) (**Figure 6B**) reveals characteristics peaks at 1692, 1756, and 1764 cm^-1^, which represent the C = O stretching in conjugated ketones (Popescu et al., 2007) and carboxylic acid groups (Schwanninger et al., 2004), alkyl esters (including the methyl ester of fatty acids), and in γ-lactone (Lievens et al., 2011), respectively. The band at 1164 cm^-1^ is typical for 5,7- dihydroxy-substituted flavonoids (Zu et al., 2012) and indicates the presence of taxifolin. It is also important to highlight the presence of the band at 1072 cm^-1^, associated with C-O deformation in primary and secondary alcohol groups of galactosyl subunits (Ghosh et al., 2015). In the S2 layer (point nr. 3), the AFM-IR spectrum shows more intense cellulose/hemicellulose bands, whereas the band at 1660 cm^-1^ is shifted to higher wavenumbers compared to the S2 layer in SW, likely due to the formation of C = O conjugated ketones. Nevertheless, the lignin band is again more intense and a shoulder at 1640-1660 cm^-1^ appears.

## Discussion

The formation of HW is linked to the occurrence of non-structural substances called extractives, which play an important role in the resistance of wood to fungal decay (Hinterstoisser et al., 2000; Schultz and Nicholas, 2000). By combining high resolution SR-FTIR with the powerful unmixing algorithm MCR-ALS, we were able to identify a component associated mainly with phenolic compounds and likely with deposition of resin acids (component III from **D**_X_ and **D**_T_, **Figures 4** and **S2**). Component III shows prominent IR bands at 1637, 1658, and 1728 cm^-1^ in the ray, tracheid lumen, and S3 layer. The band around 1637 cm^-1^ was assigned to the ketone bond in taxifolin (Ruddick and Xie, 1994; Miklečić et al., 2012; Kocábová et al., 2016; Liu et al., 2018), one of the most abundant phenolic compounds in larch wood (Giwa and Swan, 1975; Babkin et al., 2001; Ivanova et al., 2012; Zule et al., 2017) (see reference spectrum of taxifolin in **Figure S4** for comparison). The other peaks seen in the taxifolin spectrum are not seen due to overlap with the spectra of the structural wood cell wall biopolymers. The distinct, broad band 1700-1736 cm^-1^ centered at 1728 cm^-1^, is assigned to the C = O vibration of carboxylic acid groups in resin acids (Faix, 1991; Schwanninger et al., 2004). It is important to emphasize that component III appears to be more common in the ray than in tracheid cells in the SW and vice versa in the HW (see **Figures 4B** and **S3**). As described by Hillis (1987), parenchyma cells die when HW forms and the polyphenols diffuse into cell walls. The relatively higher concentration of taxifolin and resin acid mixtures in the HW cell walls likely explains its natural durability.

With the MCR-ALS multiset analysis of tangential and cross-sections, two MCR-ALS contributions were found to be directly linked to the process of HW formation in Kurile larch (components IV and V, **Figures 4** and **S3**). Both of them mainly showed lignin bands but had different spatio-temporal distributions, as well as different spectral features. Component IV was located in the CC and CML of tracheid cells in the SW, although in the transition zone it was almost exclusively present in the ray area. The other lignin contribution (Component V) was distributed in CC, CML, and in the ray cells of HW. The main spectroscopic difference between component IV and V was the appearance of the band at 1640 cm^-1^ and the simultaneous disappearance of the band at 1660 cm^-1^.The disappearance of the 1660 cm^-1^ band might be explained by a decrease in the intensity or as a drastic shift in the frequency to 1640 cm^-1^.

The decrease of the intensity of band at 1655-1660 cm^-1^ could be explained in terms of condensation reactions (Yamauchi et al., 2005) of coniferyl alcohol in lignin. By condensation, coniferyl alcohol loses the ethylenic bond which then no longer contributes to the vibration at 1660 cm^-1^. However, this process cannot explain the appearance of the 1640 cm^-1^ band. Possible coupling of the condensation reaction with the integration of new coniferyl aldehyde moieties into the lignin structure and their H-bonding to unreacted alcohols would decrease the frequency of the 1660 cm^-1^ band to 1640 cm^-1^, as described by Bock and Gierlinger (2019) and Agarwal and Reiner (2009). Another possibility for the band shift is the oxidation of coniferyl alcohol to its aldehyde and subsequent H-bonding to the carbonyl groups during the aging of the wood cells. Lastly, the appearance of taxifolin and other flavonoids may explain the appearance of the 1640 cm^-1^ band, as they contain carbonyl vibrations at 1640 cm^-1^. Since flavonoids are rich in OH-groups, they are likely to interact with lignin and cause the shift of the 1660 cm^-1^ band frequency.

From the MCR-ALS distribution maps, interpretation of the deposition pathways of the components involved during HW formation of larch was achieved. It is interesting to observe that distribution of extractives (component III from **D**_X_ and **D**_T_, see **Figures 4A** and **S3A**) is represented in all the images and can be found in the ray region, lumen, and S3 layer of tracheid cells. It may suggest that precursor molecules are present before and after the actual transition from SW to HW. This pattern is observed in Juglans-Type II HW formation. Not much is known about this mechanism, but it has been described for deciduous trees, as well as conifers, such as *Prunus*, *Platycarya*, *Eucalyptus*, and *Pseudotsuga* (Kampe and Magel, 2013). If we follow their deposition in the distribution maps of **Figures 4A** **and** **S3A**, it seems that extractives are accumulated in the ray in SW and after ray cell death, they spread to the surrounding wood tissues. We can also see how component V emerges and component IV diminishes during the HW formation process. The similar IR spectra and spatial deposition indicate that component IV corresponds to a set of precursor molecules of component V. Thus, Type II seems the reasonable mechanism of larch HW formation.

AFM-IR spectroscopy revealed variations in the composition between the tracheid and the ray regions in SW and HW (**Figures 5A, B**). The main spectral differences were found at 1108, 1456, 1648, and 1660 cm^-1^. In the HW tracheid cell walls, the band at 1108 cm^-1^, assigned to COH in plane deformation of celluloses and hemicelluloses (Schwanninger et al., 2004) and/or to aromatic C-H in plane deformation of lignin, increases. At the same time, the band at 1456 cm^-1^, related to C-H bending of methoxyl groups (Schwanninger et al., 2004) becomes broader. According to the literature, the IR band at 1108 cm^-1^ could appear because of cross-linking reactions of –OH groups of cellulose/hemicellulose with phenolic compounds at the cell wall level (Wang et al., 2016), resulting in an increase in the hardness of the wood cell walls. The most important difference is again the appearance of the band at 1648 cm^-1^ and the absence of the band at 1660 cm^-1^ in the HW tracheid cell walls. As mentioned earlier, the band at 1655 cm^-1^ could be mainly present because of C = O and C = C groups in coniferyl aldehyde and coniferyl alcohol structures of lignin. Hence, it is likely that the reduction of band intensity may be attributable mainly to condensation reactions of lignin molecules or oxidative alteration of lignin.

It is well known that lignin condensation makes lignocellulosic biomass more recalcitrant, mainly due to limiting the accessibility to the polysaccharides in the cell wall (Li et al., 2016). Additionally, generation of new carbonyl groups by oxidation, as discussed above, may increase non-productive binding of cellulases or enzyme inhibition *via* chelation of metal co-factors (Li et al., 2016), thereby starving the fungus. The attachment of other, potentially fungitoxic or antioxidant phenolics, i.e. flavonoids, to these newly formed reaction sites are other possibilities that lignin modification would allow for. These steric or chemical inhibitory effects are important for understanding the durability of heartwood (Valette et al., 2017).

The AFM-IR spectra of the ray also show differences between SW and HW (**Figures 6A, B**).

For example, the appearance of the band around 1680-1692 cm^-1^ suggests the presence of conjugated ketones and carboxylic acids, such as resin acids, in the HW ray and its surrounding cell wall tracheid. The presence of taxifolin inside the ray is supported by the presence of the band at 1164 cm^-1^ (a characteristic band for 5,7- dihydroxysubstituted flavonoids) (Zu et al., 2012).

We can also observe an intense C = O stretching vibration at 1764 cm^-1^ and around 1750 cm^-1^ (see **Figure 6B**), indicating the presence of γ-lactones and alkyl esters, including the methyl esters of fatty acids (Lievens et al., 2011). Prominent bands that appear at 1072 and 1728 cm^-1^ are assigned to C-O deformation in primary and secondary alcohol groups of galactosyl- and carbonyl of carboxylic groups.

The interpretation of the spectra is complex since mixtures of chemical compounds are present throughout the plant tissue, i.e spectra of pure chemical constituents are rarely possible to obtain, neither by use of high spatial resolution (as in AFM-IR), nor by use of MCR-ALS modelling. Consequently, comparing to a spectral data base with the most common components present in plant cell walls might help to further identify the chemical compounds, but it would most likely not lead to conclusive results. AFM-IR is presented in this study as a potent technique to further characterize plant cell wall components because of its higher spatial resolution than SR-IR imaging, and because spectra could be obtained for a broader range of wavenumbers. However, measurements of a more comprehensive sample set would be necessary in order to not simply illustrate the technique but obtain representative results.

## Conclusions

MCR-ALS multiset analysis on sets of SR-FTIR images collected across the HW formation zone of Kurile larch provided a cellular level description of the components involved in HW formation. In particular, the IR resolved spectral signatures and comparison with IR reference spectra from the literature allowed us to identify taxifolin, one of the most abundant extractive in larch, in rays as well as in the lumen and S3 cell wall layer of adjacent tracheids. Moreover, refolding of the concentration profiles to the original image formats allowed us to see that one initial phenolic lignan contribution (component IV) was present in the SW, while a second somewhat similar contribution (component V) emerged in the transition zone and continued in the HW. Our interpretation of this result is that component IV is a set of precursor molecules for component V. Such a pattern is characteristic for Type II heartwood formation, also called Juglans-type. The main spectroscopic difference between component IV and V was the appearance of the band at 1640 cm^-1^ and the simultaneous disappearance of the band at 1660 cm^-1^. We hypothesize that the disappearance of the 1660 cm^-1^ band may be attributable mainly to condensation reactions of lignin/lignan molecules or oxidative alteration of lignin. Lignin condensation reactions are known to make lignin more recalcitrant. Generation of new carbonyl groups by oxidation of coniferyl alcohol to coniferyl aldehyde could also help explain both the peak shift and the resistance against fungal attack of Kurile larch HW.

AFM-IR has been proven to be a powerful technique to study the nanoscale compositional variations between the cell wall, CML, and the ray of SW and HW of larch. The AFM-IR results confirmed the trends observed in the SR-FTIR image analysis and provided more detail about the plant cell wall composition as spectra were obtained for a broader spectral range. Conjugated ketones and carboxylic acids accompanied with the presence of γ-lactone and alkyl ester were also found in the HW rays. Finally, AFM-IR spectra proposed the existence of cross-linked reactions of cellulose/hemicelluloses with phenol compounds at the cell wall level in HW.

## Data Availability Statement

The datasets generated for this study are available on request to the corresponding author.

## Author Contributions

All the authors discussed the results and contributed to the final manuscript. SP, SF, RO, AG-S, TK, AJ and LT carried out the SR-FTIR experiment and SJ performed the AFM-IR measurements.

## Funding

The research was funded by VILLUM FONDEN through project 12404. RO, AG-S and AJ also acknowledge funding from the Spanish government through project CTQ2015–66254-C2-2-P.

## Conflict of Interest

The authors declare that the research was conducted in the absence of any commercial or financial relationships that could be construed as a potential conflict of interest.

The handling editor is currently co-organizing a Research Topic with one of the authors, LT, and confirms the absence of any other collaboration.

## Supplementary Material

Figure S1 Schematic diagram of AFM-IR setup. IR laser irradiates the sample which then expands. The expansion of the sample is detected by the AFM tip which deflects the whole AFM cantilever. The deflection is then monitored by tracking the movements of the AFM laser shone on the cantilever.

Figure S2
**(A)** Representation of the area selection of the lumen, cell wall, and ray. **(B)** Average spectra of the cell wall selected for each of the cross section images collected across the heartwood formation zone. **(C)** Zoom of the spectral range 1200 cm^-1^ to 1800 cm^-1^ of the average spectra of the cell wall selected for each of the cross section. The spectra gradually change from blue (sapwood) to red (heartwood) color.

Figure S3 MCR-ALS results of the multiset structure formed by a series of tangential section images. **(A)** distribution maps of components involved in the heartwood formation of Kurile larch. Each line of maps represents the resolved maps of all constituents for a particular sample. Each column of maps represents the distribution map of a particular chemical constituent in all samples analyzed. Distribution maps use a gradual color scale where yellow color refers to large concentration values and blue color to small values. **(B)** Related pure spectra.

Figure S4 FTIR spectrum of taxifolin crystal according to (Liu et al., 2018).

## References

[B1] Agarwal U. P. Reiner R. S. (2009). Near-IR Surface-Enhanced Raman Spectrum of Lignin † A. J. Raman Spectrosc. 40, 1527–1534. 10.1002/Jrs.2294

[B2] Babkin V. A. Ostroukhova L. A. Malkov Y. A. Babkin D. V. Onuchina N. A. Ivanova S. Z. (2001). Isolation of biologically active compounds from larch wood. Int. Symp. Wood Pulping Chem. 2, 119–122.

[B3] Belt T. Keplinger T. Hänninen T. Rautkari L. (2017). Cellular level distributions of Scots pine heartwood and knot heartwood extractives revealed by Raman spectroscopy imaging. Nice, France. Ind. Crops Prod. 108, 327–335. 10.1016/j.indcrop.2017.06.056

[B4] Bergström B. Gustafsson G. Gref R. Ericsson A. (1999). Seasonal changes of pinosylvin distribution in the sapwood/heartwood boundary of Pinus sylvestris. Trees - Struct. Funct. 14, 65–71. 10.1007/s004680050210

[B5] Bito N. Nakada R. Fukatsu E. Matsushita Y. Fukushima K. Imai T. (2011). Clonal variation in heartwood norlignans of Cryptomeria japonica: Evidence for independent control of agatharesinol and sequirin C biosynthesis. Ann. For. Sci. 68, 1049–1056. 10.1007/s13595-011-0118-7

[B6] Bock P. Gierlinger N. (2019). Infrared and Raman spectra of lignin substructures: Coniferyl alcohol, abietin, and coniferyl aldehyde. Raman Spectrosc. 50, 778–792. 10.1002/jrs.5588 PMC6602882 31263319

[B7] Bro R. de Jong S. (1997). A fast non-negativity-constrained least squares algorithm. J. Chemom. 11, 393–401.

[B8] Burčová Z. Kreps F. Greifová M. Jablonský M. Ház A. Schmidt Š. (2018). Antibacterial and antifungal activity of phytosterols and methyl dehydroabietate of Norway spruce bark extracts. J. Biotechnol. 282, 18–24. 10.1016/J.JBIOTEC.2018.06.340 29940188

[B9] Burtin P. Jay-Allemand C. Charpentier J. P. Janin G. (1998). Natural wood colouring process in Juglans sp. (J. nigra, J. regia and hybrid J. nigra 23 x J. regia) depends on native phenolic compounds accumulated in the transition zone between sapwood and heartwood. Trees - Struct. Funct. 12, 258–264. 10.1007/PL00009716

[B10] Bush D. McCarthy K. Meder R. (2011). Genetic variation of natural durability traits in Eucalyptus cladocalyx (sugar gum). Ann. For. Sci. 68, 1057–1066. 10.1007/s13595-011-0121-z

[B11] Cao G. Sofic E. Prior R. L. (1997). Antioxidant and Prooxidant Behavior of Flavonoids: Structure-Activity Relationships. Free Radic. Biol. Med. 22, 749–760. 10.1016/S0891-5849(96)00351-6 9119242

[B12] Colom X. Carrillo F. Nogue F. Garriga P. (2003). Structural analysis of photodegraded wood by means of FTIR spectroscopy. Polym. Degrad. Stab. 80, 543–549. 10.1016/S0141-3910(03)00051-X

[B13] Compounds M. Hergert H. L. (1960). Infrared Spectra of Lignin and Related Compounds. II. Conifer Lignin and. J. Org. Chem. 25, 405–413. 10.1021/jo01073a026

[B14] Déjardin A. Laurans F. Arnaud D. Breton C. Pilate G. Leplé J.-C. (2010). Wood formation in Angiosperms. C. R. Biol. 333, 325–334. 10.1016/J.CRVI.2010.01.010 20371107

[B15] Dazzi A. Prater C. B. (2017). AFM-IR: Technology and applications in nanoscale infrared spectroscopy and chemical imaging. Chem. Rev. 117, 5146–5173. 10.1021/acs.chemrev.6b00448 27958707

[B16] Dazzi A. Prazeres R. Ortega J. M. (2005). Local infrared microspectroscopy with subwavelength spatial resolution with an atomic force microscope tip used as a photothermal sensor. Opt. Lett. 30, 2388–2390. 10.1364/OL.30.002388 16196328

[B17] Dazzi A. Prater C. B. Hu Q. Chase D. B. Rabolt J. F. Marcott C. (2012). AFM-IR: Combining atomic force microscopy and infrared spectroscopy for nanoscale chemical characterization. Appl. Spectrosc. 66, 1365–1384. 10.1366/12-06804 23231899

[B18] de Juan A. Tauler R. (2006). Multivariate Curve Resolution (MCR) from 2000: progress in concepts and applications. Crit. Rev. Anal. Chem. 36, 163–176. 10.1080/10408340600970005

[B19] de Juan A. Tauler R. Dyson R. Marcolli C. Rault M. Maeder M. (2004). Spectroscopic imaging and chemometrics: a powerful combination for global and local sample analysis. TrAC Trends Anal. Chem. 23, 70–79. 10.1016/S0165-9936(04)00101-3

[B20] Dellus V. Scalbert A. Janin G. (1997). Polyphenols and colour of Douglas fir heartwood. Holzforschung 51, 291–295. 10.1515/hfsg.1997.51.4.291

[B21] Eilers P. H. C. (2004). Parametric time warping. Anal. Chem. 76, 404–411. 10.1021/ac034800e 14719890

[B22] Fackler K. Stevanic S. J. Ters T. Hinterstoisser B. Schwanninger M. Salmén L. (2010). Localisation and characterisation of incipient brown-rot decay within spruce wood cell walls using FT-IR imaging microscopy. Enzyme Microb. Technol. 47 (6), 257–267. 10.1016/j.enzmictec.2010.07.009 21052475 PMC2954293

[B23] Faix O. (1991). Classification of Lignins from different botanical origins by FT-IR spectroscopy. Holzforschung 45, 21–28. 10.1515/hfsg.1991.45.s1.21

[B24] Farber C. Wang R. Chemelewski R. Mullet J. Kurouski D. (2019). Nanoscale structural organization of plant epicuticular wax probed by atomic force microscope infrared spectroscopy. Anal. Chem. 91, 2472–2479. 10.1021/acs.analchem.8b05294 30624904

[B25] Felhofer M. Prats-Mateu B. Bock P. Gierlinger N. (2018). Antifungal stilbene impregnation: transport and distribution on the micron-level. Tree Physiol. 38, 1526–1537. 10.1093/treephys/tpy073 29992254 PMC6198867

[B26] Felten J. Hall H. Jaumot J. Tauler R. de Juan A. Gorzsás A. (2015). Vibrational spectroscopic image analysis of biological material using multivariate curve resolution-alternating least squares (MCR-ALS). Nat. Protoc. 10, 217–240. 10.1038/nprot.2015.008 25569330

[B27] Fengel D. Wegener G. (1989). Wood: Chemistry, Ultrastructure,Reactions (Berlin-New York: Walter de Gryter).

[B28] Ghosh T. Basu A. Adhikari D. Roy D. (2015). Antioxidant activity and structural features of Cinnamomum zeylanicum. 3 Biotech 5, 939–947. 10.1007/s13205-015-0296-3 PMC4624148 28324396

[B29] Gierlinger N. Schwanninger M. Hinterstoisser B. Wimmer R. (2002). Rapid determination of heartwood extractives in Larix sp. by means of Fourier transform near infrared spectroscopy. J. Near Infrared Spectrosc. 10, 203–214. 10.1255/jnirs.336

[B30] Gierlinger N. Jacques D. Grabner M. Wimmer R. Schwanninger M. Rozenberg P. (2004). Colour of larch heartwood and relationships to extractives and brown-rot decay resistance. Trees - Struct. Funct. 18, 102–108. 10.1007/s00468-003-0290-y

[B31] Giwa S. Swan E. (1975). Heartwood extractives of a western larch tree (Larix occidentalis Nutt.). Wood Fiber 7, 216–221.

[B32] Golub G. H. Reinsch C. (1970). Singular value decomposition and least squares solutions. Numer. Math. 14, 403–420. 10.1007/BF02163027

[B33] Gorsás A. Stenlund H. Persson P. Trygg J. (2011). Cell-specific chemotyping and multivariate imaging by combined FT-IR microspectroscopy and orthogonal projections to latent structures (OPLS) analysis reveals the chemical landscape of secondary xylem. Plant J. 66, 903–914. 10.1111/j.1365-313X.2011.04542.x 21332846

[B34] Higuchi T. (1997). Biochemistry and Molecular Biology of Wood. Ed. Higuchi T., Springer Series in Wood Science (Berlin, Heidelberg: Springer).

[B35] Hillis W. (1985). “Ocurrence of extractives in wood tissue,” in Biosynthesis and biodegradation of wood components. Ed. Higuchi T. (Orlando: Academic Press, Inc.) 209–228.

[B36] Hillis W. (1987). Heartwood and Tree Exudates, Springer Series in Wood Science (Berlin: Springer-Verlag).

[B37] Hinterstoisser B. Stefke B. Schwanninger M. (2000). Wood: raw material-materiak-source of energy for the future. Lignovisionen 2, 29–36. 10.1016/S0924-2031(99)00063-6

[B38] Imai T. Tanabe K. Kato T. Fukushima K. (2005). Localization of ferruginol, a diterpene phenol, in Cryptomeria japonica heartwood by time-of-flight secondary ion mass spectrometry. Planta 221, 549–556. 10.1007/s00425-004-1476-2 15856284

[B39] Ivanova S. Z. Gorshlov A. G. Kuzmin A. V. Gordienko I. I. Babkin V. A. (2012). Phenolic compounds of Siberian and Dahurian larch phloem. Russ. J. Bioorg. Chem. 38, 769–774. 10.1134/S1068162012070096

[B40] Janik E. Bednarska J. Zubik M. Puzio M. Luchowski R. Grudzinski W. (2013). Molecular architecture of plant thylakoids under physiological and light stress conditions: a study of lipid-light-harvesting complex II model membranes. Plant Cell 25, 2155–2170. 10.1105/tpc.113.113076 23898030 PMC3723618

[B41] Jaumot J. Gargallo R. de Juan A. Tauler R. (2005). A graphical user-friendly interface for MCR-ALS: a new tool for multivariate curve resolution in MATLAB. Chemometr. Intell. Lab. Syst. 76, 101–110. 10.1016/j.chemolab.2004.12.007

[B42] Kampe A. Magel E. (2013). “New insights into heartwood and heartwood formation,” in Cellular Aspects of Wood Formation. Ed. Fromm J. (Berlin: Springer), 71–95.

[B43] Keeling C. I. Bohlmann J. (2006). Diterpene resin acids in conifers. Phytochemistry 67, 2415–2423. 10.1016/J.PHYTOCHEM.2006.08.019 16996548

[B44] Kocábová J. Fiedler J. Degano I. Sokolová R. (2016). Electrochimica Acta Oxidation mechanism of fl avanone taxifolin. Electrochemical and spectroelectrochemical investigation. Electrochim. Acta 187, 358–363. 10.1016/j.electacta.2015.11.077

[B45] Kwon M. Davin L. B. Lewis N. G. (2001). *In situ* hybridization and immunolocalization of lignan reductases in woody tissues: implications for heartwood formation and other forms of vascular tissue preservation. Phytochemistry 57, 899–914. 10.1016/S0031-9422(01)00108-X 11423140

[B46] Lawther J. M. Sun R. Banks W. B. (1996). Fractional Characterization of Wheat Straw Lignin Components by Alkaline Nitrobenzene Oxidation and FT-IR Spectroscopy. Appl. Spectrosc. 44, 1241–1247. 10.1021/jf9502764

[B47] Li M. Pu Y. Ragauskas A. J. (2016). Current understanding of the correlation of lignin structure with biomass recalcitrance. Front. Chem. 4, 1–8. 10.3389/fchem.2016.00045 27917379 PMC5114238

[B48] Lievens C. Mourant D. He M. Gunawan R. Li X. Li C. Z. (2011). An FT-IR spectroscopic study of carbonyl functionalities in bio-oils. Fuel 90, 3417–3423. 10.1016/j.fuel.2011.06.001

[B49] Liu Z. Wei M. Cui G. Yang X. Gu H. Yang L. (2018). Optimization of arabinogalactan and taxifolin extraction process from Dahurian larch (Larix gmelinii) and evaluation of the effects on activities of α-amylase, α-glycosidase, and pancreatic lipase in vitro. J. Food Biochem. 42, 1–14. 10.1111/jfbc.12607

[B50] Magel E. Jay-Allemand C. Ziegler H. (1994). Formation of heartwood substances in the stemwood of Robinia pseudoacacia L. II. Distribution of nonstructural carbohydrates and wood extractives across the trunk. Trees 8, 165–171. 10.1007/BF00196843

[B51] Mayer I. Koch G. Puls J. (2006). Topochemical investigations of wood extractives and their influence on colour changes in American black cherry (Prunus serotina Borkh.). Holzforschung 60, 589–594. 10.1515/HF.2006.100

[B52] Miklečić J. Španić N. Jirouš-Rajković V. (2012). Wood color changes by ammonia fuming. BioResources 7, 3767–3778.

[B53] Monties B. (1991). Plant cell walls as fibrous lignocellulosic composites: relations with lignin structure and function. Anim. Feed Sci. Technol. 32, 159–175. 10.1016/0377-8401(91)90019-O

[B54] Nair M. N. Shah J. Pandalai R. C. (1981). Wood anatomy and histochemical changes of sapwood during heartwood formation in Bridelia retusa Spreng. Proc. Plant Sci. 90, 425–433. 10.1007/BF03052940

[B55] Nisula L. (2018). Wood extractives in conifers: a study of stemwood and knots of industrially important species. Åbo, Finland: Åbo Akademi University Press. 372. Available at: https://www.doria.fi/handle/10024/149340.

[B56] Pereira L. Flores-Borges D. N. A. Bittencourt P. R. L. Mayer J. L. S. Kiyota E. Araújo P. (2018). Infrared nanospectroscopy reveals the chemical nature of pit membranes in water-conducting cells of the plant xylem. Plant Physiol. 177, 1629–1638. 10.1104/pp.18.00138 29871981 PMC6084671

[B57] Piqueras S. Duponchel L. Offroy M. Jamme F. Tauler R. de Juan A. (2013). Chemometric strategies to unmix information and increase the spatial description of hyperspectral images: a single-cell case study. Anal. Chem. 85, 6303–6311. 10.1021/ac4005265 23697511

[B58] Piqueras S. Duponchel L. Tauler R. de Juan A. (2014). Monitoring polymorphic transformations by using *in situ* Raman hyperspectral imaging and image multiset analysis. Anal. Chim. Acta 819, 15–25. 10.1016/j.aca.2014.02.027 24636406

[B59] Piqueras S. Krafft C. Beleites C. Egodage K. von Eggeling F. Guntinas-Lichius O. (2015). Combining multiset resolution and segmentation for hyperspectral image analysis of biological tissues. Anal. Chim. Acta 881, 24–36. 10.1016/j.aca.2015.04.053 26041517

[B60] Pomar F. Merino F. Barceló A. R. (2002). O -4-Linked coniferyl and sinapyl aldehydes in lignifying cell walls are the main targets of the Wiesner (phloroglucinol-HCl) reaction. Protoplasma 220, 17–28. 10.1007/s00709-002-0030-y 12417933

[B61] Popescu C. Popescu M. Singurel G. Vasile C. Argyropoulos D. S. Willfor S. (2007). Spectral characterization of eucalyptus wood. Appl. Spectrosc. 61, 1168–1177. 10.1366/000370207782597076 18028695

[B62] Popescu C. Popescu M. Vasile C. (2010). Characterization of fungal degraded lime wood by FT-IR and 2D IR correlation spectroscopy Characterization of fungal degraded lime wood by FT-IR and 2D IR correlation spectroscopy. Microchem. J. 95, 377–387. 10.1016/j.microc.2010.02.021

[B63] Romeo M. Mohlenhoff B. Diem M. (2006). Infrared micro-spectroscopy of human cells: causes for the spectral variance of oral mucosa (buccal) cells. Vib. Spectrosc. 42, 9–14. 10.1016/j.vibspec.2006.04.009.Infrared 19750140 PMC2742430

[B64] Ruddick J. N. R. Xie C. (1994). Why does Douglas-fir heartwood turn black when treated with ammoniacal copper preservatives? For. Prod. J. 44, 57–61.

[B65] Saito K. Mitsutani T. Imai T. Matsushita Y. Yamamoto A. Fukushima K. (2008). Chemical differences between sapwood and heartwood of Chamaecyparis obtusa detected by ToF-SIMS. Appl. Surf. Sci. 255, 1088–1091. 10.1016/J.APSUSC.2008.05.145

[B66] Savitzky A. Golay M. J. E. (1964). Smoothing and differentiation of data by simplified least squares procedures. Anal. Chem. 36, 1627–1639. 10.1021/ac60214a047 22324618

[B67] Schultz T. P. Nicholas D. D. (2000). Naturally durable heartwood: evidence for a proposed dual defensive function of the extractives. Phytochemistry 54, 47–52. 10.1016/S0031-9422(99)00622-6 10846746

[B68] Schwanninger M. Rodrigues J. C. Pereira H. Hinterstoisser B. (2004). Effects of short-time vibratory ball milling on the shape of FT-IR spectra of wood and cellulose. Vib. Spectrosc. 36, 23–40. 10.1016/j.vibspec.2004.02.003

[B69] Shi J. Xing D. Li J. (2012). FTIR Studies of the changes in wood chemistry from wood (1640 C = O).pdf. Energy Proc. 16, 758–762. 10.1016/j.egypro.2012.01.122

[B70] Stewart D. Wilson H. Hendra P. Morrison M. (1995). Fourier-transform infrared and raman spectroscopic study of biochemical and chemical treatments of oak wood (Quercus rubra) and barley (Hordeum vulgare) Straw. J. Agric. Food. Chem. 43, 2219–2225. 10.1021/jf00056a047

[B71] Tauler R. Smilde A. Kowalski B. (1995). Selectivity, local rank, three-way data analysis and ambiguity in multivariate curve resolution. J. Chemometr. 9, 31–58. 10.1016/0169-7439(95)00047-X

[B72] Tauler R. Maeder M. de Juan A. (2009). “Multiset Data Analysis: Extended Multivariate Curve Resolution,” in Comprehensive Chemometrics: Chemical and Biochemical Data Analysis. Eds. Brown S. D. Tauler R. Walczak B. (Amsterdam, The Netherlands: Elsevier), 473–506. Available at: http://www.iasbs.ac.ir/chemistry/chemometrics/history/11th/multisetdataanalysis.pdf [Accessed February 16, 2015].

[B73] Taylor A. M. Gartner B. L. Morrell J. J. (2002). Heartwood formation and natural durability - A review. Wood Fiber Sci. 34, 587–611.

[B74] Tomppo L. Tiitta M. Laakso T. Harju A. Venäläinen M. Lappalainen R. (2011). Study of stilbene and resin acid content of Scots pine heartwood by electrical impedance spectroscopy (EIS). Holzforschung 65, 643–649. 10.1515/HF.2011.111

[B75] Traoré M. Kaal J. Martínez Cortizas A. (2018). Differentiation between pine woods according to species and growing location using FTIR-ATR. Wood Sci. Technol. 52, 487–504. 10.1007/s00226-017-0967-9 29497215 PMC5816091

[B76] Umesh P. Rajai H. (2010). “Vibrational Spectroscopy,” in Lignin and Lignans: Advances in Chemistry. Eds. Heitner C. Dimmel D. Schmidt J. A. (Boca Raton, Florida: CRC Press Taylor&Francis group), 104–129.

[B77] Umesh P. James D. Sally A. (2011). FT – Raman investigation of Milled-Wood Lignins: softwood, hardwood, and chemically modified black spruce lignins. J. Wood Chem. Technol. 31, 324–344. 10.1080/02773813.2011.562338

[B78] Valette N. Perrot T. Sormani R. Gelhaye E. Morel-Rouhier M. (2017). Antifungal activities of wood extractives. Fungal Biol. Rev. 31, 113–123. 10.1016/j.fbr.2017.01.002

[B79] Wang Q. Kretlow A. Beekes M. Naumann D. Miller L. (2005). *In situ* characterization of prion protein structure and metal accumulation in scrapie-infected cells by synchrotron infrared and X-ray imaging. Vib. Spectrosc. 38, 61–69. 10.1016/j.vibspec.2005.02.023

[B80] Wang X. Deng Y. Li Y. Kjoller K. Roy A. Wang S. (2016). *In situ* identification of the molecular-scale interactions of phenol-formaldehyde resin and wood cell walls using infrared nanospectroscopy. RSC Adv. 6, 76318–76324. 10.1039/c6ra13159j

[B81] Weiland J. J. Guyonnet R. (2003). Study of chemical modifications and fungi degradation of thermally modified wood using DRIFT spectroscopy. Holz als Roh und Werkstoff. 61, 216–220. 10.1007/s00107-003-0364-y

[B82] Willför S. Holmbom B. (2004). Isolation and characterisation of water soluble polysaccharides from Norway spruce and Scots pine. Wood Sci. Technol. 38, 173–179. 10.1007/s00226-003-0200-x

[B83] Windeisen E. Wegener G. Lesnino G. Schumacher P. (2002). Investigation of the correlation between extractives content an natural durability in 20 cultivated larch trees. Holz als Roh - und Werkst. 60, 373–374. 10.1007/s00107-002-0314-0

[B84] Windig W. Guilment J. (1991). Interactive self-modeling mixture analysis. Anal. Chem. 63, 1425–1432. 10.1021/ac00014a016

[B85] Yamauchi S. Iijima Y. Doi S. (2005). Spectrochemical characterization by FT-Raman spectroscopy of wood heat-treated at low temperatures: Japanese larch and beech. Japan Wood Res. Soc 51, 498–506. 10.1007/s10086-004-0691-6

[B86] Zu S. Yang L. Huang J. Ma C. Wang W. Zhao C. (2012). Micronization of Taxifolin by supercritical antisolvent process and evaluation of radical scavenging activity. Int. J. Mol. Sci. 13, 8869–8881. 10.3390/ijms13078869 22942740 PMC3430271

[B87] Zule J. Čufar K. Tišler V. (2015). Lipophilic Extractives in Heartwood of European Larch (Larix decidua Mill.). Drv. Ind. 66, 305–313. 10.5552/drind.2015.1442

[B88] Zule J. Čufar K. Tišler V. (2017). Hydrophilic Extractives in Heartwood of European Larch (Larix decidua Mill.). Drv. Ind. 67, 363–370. 10.5552/drind.2016.1618

