# Supplementary figures and images for: Understanding the Formation of Heartwood in Larch Using Synchrotron Infrared Imaging Combined With Multivariate Analysis and Atomic Force Microscope Infrared Spectroscopy
Source: Front Plant Sci. 2020 Feb 3;10:1701. doi: 10.3389/fpls.2019.01701 (PMC7008386; doi:10.3389/fpls.2019.01701)

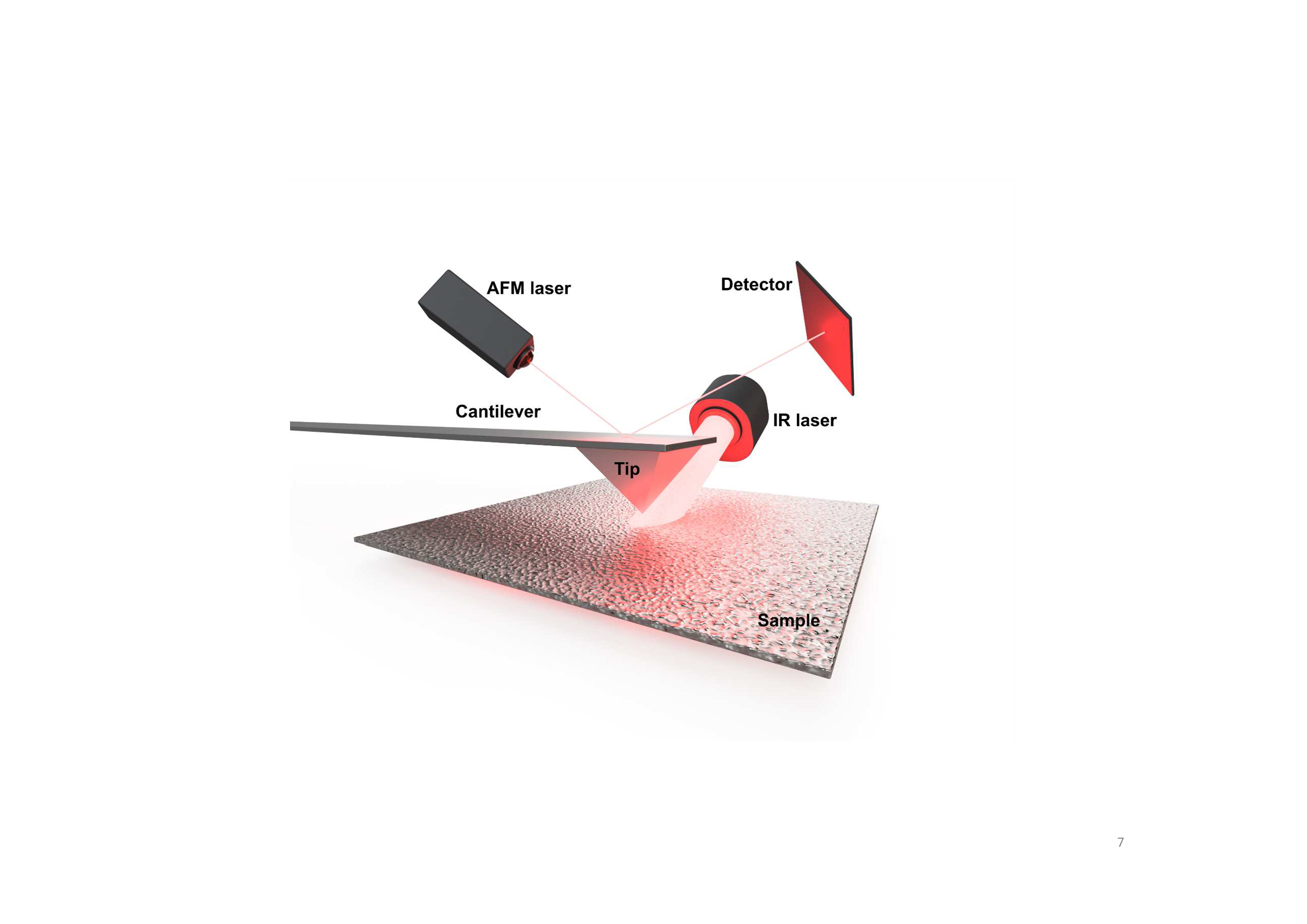

Supplement: Figure S1 — Schematic diagram of AFM-IR setup. IR laser irradiates the sample which then expands. The expansion of the sample is detected by the AFM tip which deflects the whole AFM cantilever. The deflection is then monitored by tracking the movements of the AFM laser shone on the cantilever. [file Image_1.tiff]

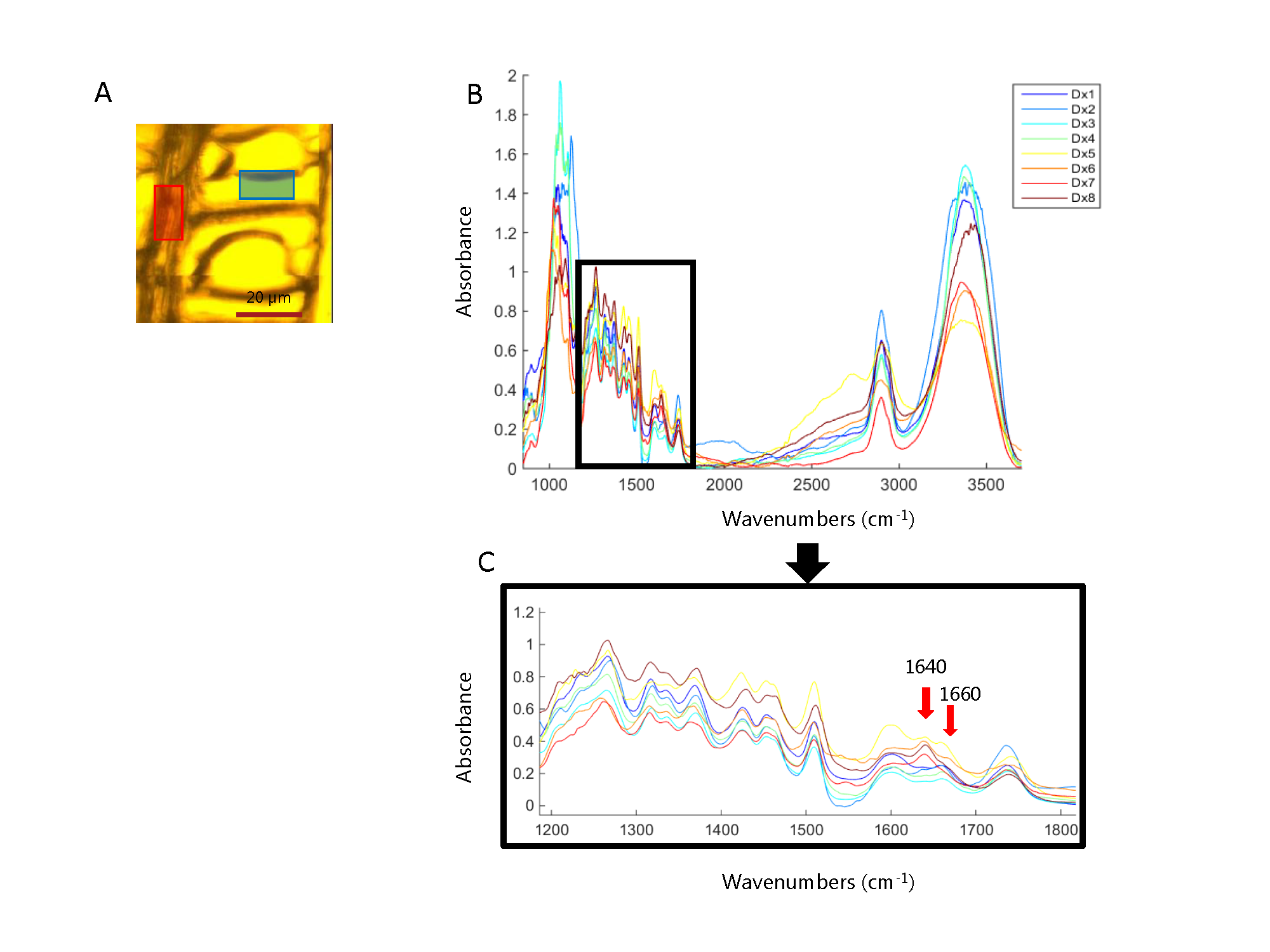

Supplement: Figure S2 — (A) Representation of the area selection of the lumen, cell wall, and ray. (B) Average spectra of the cell wall selected for each of the cross section images collected across the heartwood formation zone. (C) Zoom of the spectral range 1200 cm-1 to 1800 cm-1 of the average spectra of the cell wall selected for each of the cross section. The spectra gradually change from blue (sapwood) to red (heartwood) color. [file Image_2.tif]

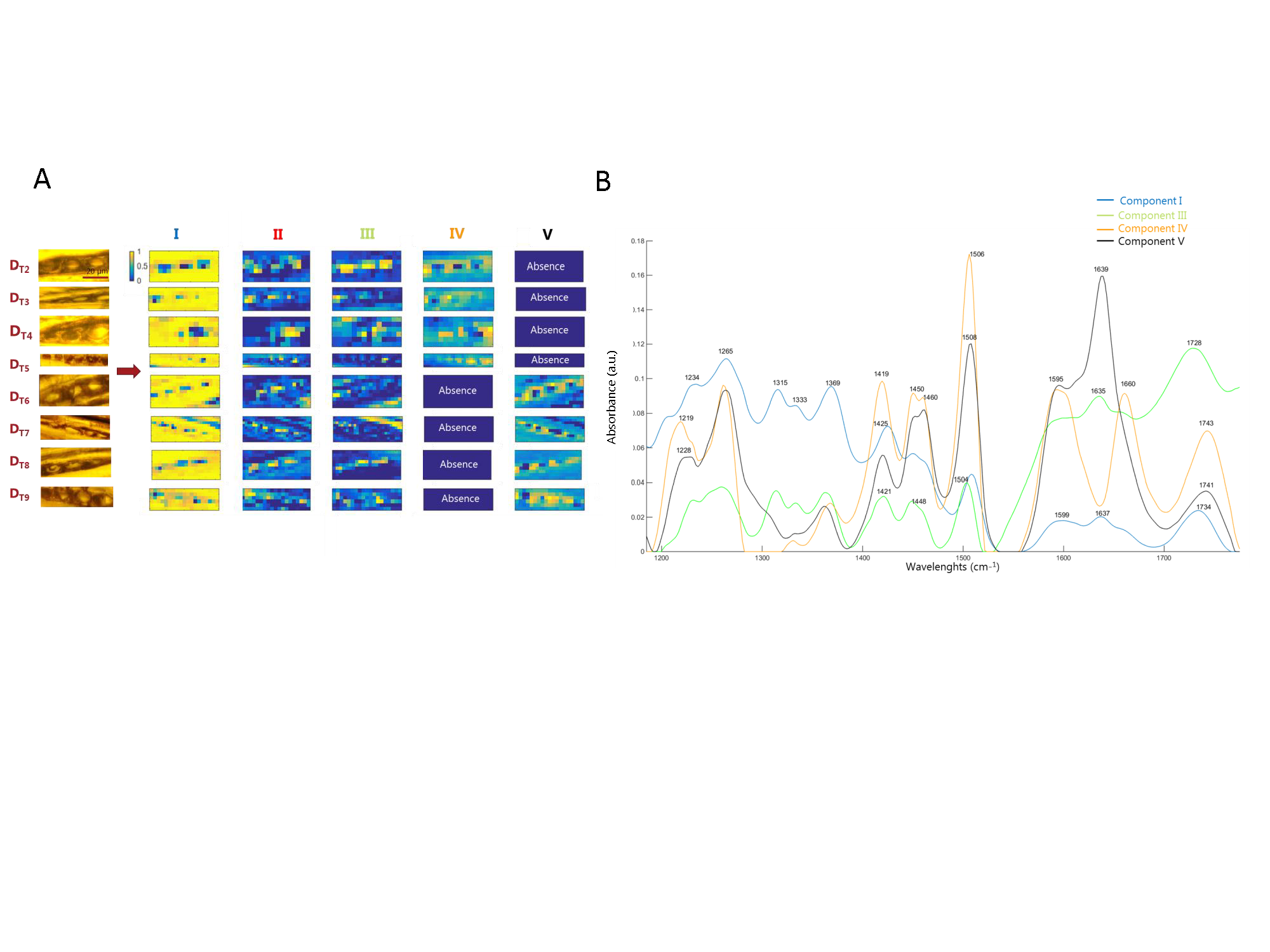

Supplement: Figure S3 — MCR-ALS results of the multiset structure formed by a series of tangential section images. (A) distribution maps of components involved in the heartwood formation of Kurile larch. Each line of maps represents the resolved maps of all constituents for a particular sample. Each column of maps represents the distribution map of a particular chemical constituent in all samples analyzed. Distribution maps use a gradual color scale where yellow color refers to large concentration values and blue color to small values. (B) Related pure spectra. [file Image_3.tiff]

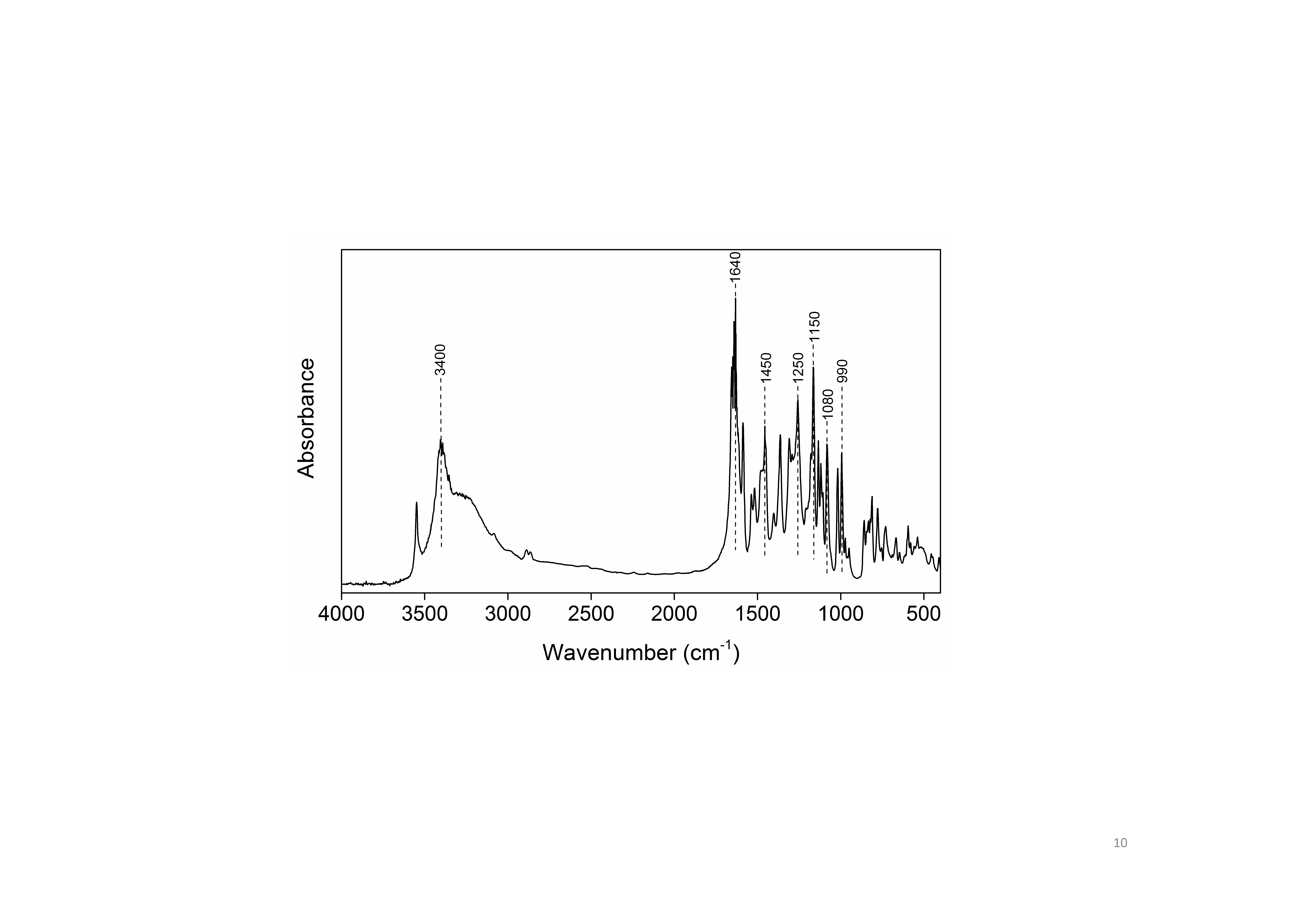

Supplement: Figure S4 — FTIR spectrum of taxifolin crystal according to (Liu et al., 2018). [file Image_4.tiff]
